# Physico-Chemical, Microbiological and Sensory Characteristics of Cabra del Guadarrama Cheese and Other Cheeses from Different Spanish Autochthonous Goat Breeds

**DOI:** 10.3390/foods14132368

**Published:** 2025-07-03

**Authors:** Teresa Herrera, Aida Pérez-Baltar, Laura Ortiz, Pablo Letón, Eugenio Miguel

**Affiliations:** Área de Investigación Agroalimentaria, Instituto Madrileño de Investigación y Desarrollo Rural, Agrario y Alimentario (IMIDRA), Alcalá de Henares, 28805 Madrid, Spain; teresa.herrera.rodriguez@madrid.org (T.H.); aida.perez@madrid.org (A.P.-B.); lauraorba@gmail.com (L.O.); pablo.leton@madrid.org (P.L.)

**Keywords:** goat cheese, autochthonous breeds, artisanal, raw milk, sensory, Cabra del Guadarrama

## Abstract

Physico-chemical analyses, fatty acid profiles, microbiological analyses and sensory characteristics (assessed by trained judges and by consumers) were carried out on four types of goat cheese produced in artisanal cheese factories using raw milk from different autochthonous Spanish goat breeds (Malagueña, Florida, Murciano-Granadina and Cabra del Guadarrama). The cheeses studied were different in fat, protein, salt, pH, moisture, acidity and color due to the different production technologies and the properties of the milk of each breed. Saturated fatty acids (SFAs) were the predominant fatty acids in all the goat milk cheeses studied. Cabra del Guadarrama Cheese (CGC) and Malagueña Cheese (MC) showed an n-6/n-3 ratio < 4, and MC was found to have the lowest atherogenic and thrombogenic indices. CGC had a lower fat content than the rest of the cheeses studied. The fatty acid profile of CGC was more similar to MC than to Florida Cheese (FC) and Murciano-Granadina Cheese (MGC). MGC had a higher atherogenic and thrombogenic index, a higher PUFA n-6/n-3 ratio than the rest of the cheeses studied and a higher fat content. Therefore, CGC, MC and FC had a healthier lipid profile than MGC. The texture properties of CGC and FC were more similar to each other than to those of MC and MGC (harder cheeses). Sensory analyses performed with trained judges were in accordance with instrumental texture parameters. Consumer acceptability was similar for all the cheeses under blind conditions and only under informed conditions did consumers score FC significantly higher than MGC. In a rank test FC was chosen as the better cheese for a greater number of consumers both in blind and in informed conditions. The provision of information improved the overall liking of Cabra del Guadarrama Cheese and worsened that of Murciano-Granadina Cheese. The high quality of the cheeses confirms the need to raise consumer awareness of autochthonous goat breeds to increase the consumption of these products in order to contribute to the preservation of these breeds.

## 1. Introduction

The goat sector represents an important segment of livestock farming. Spain is one of the main producers of goat milk in the European Union, although this only represents 2.15% of the final livestock production [1].

Spain has a significant livestock heritage. Its geographic location, diversity of ecosystems, climate, landscapes and cultural traditions have resulted in a huge variety of autochthonous livestock breeds that are well-adapted to their production environments and provide unique products of an unbeatable quality and with added value for society [2]. Unfortunately, autochthonous breeds of sheep and goat have suffered a decline in Spain in recent decades in favor of other breeds due to the greater productivity of foreign breeds. The preservation of local autochthonous breeds is a strategical instrument to conserve traditional products. Autochthonous breeds, through natural selection, are well-adapted to their particular environments and they are more resistant to diseases, being able to produce in harsher conditions [3]. In Spain, there are 22 autochthonous breeds of goat in total. Most goat milk is destined for the artisanal production of dairy products such as cheese and curds. Andalusia has a wide variety of excellent-quality cheeses linked to the territory of origin which are made in small, artisanal cheese factories with goat milk from native Andalusian breeds (Malagueña, Murciano-Granadina, Florida, Payoya, Cabra Blanca Andaluza and Cabra Negra Serrana). Andalusia is the main Spanish goat-milk-producing region, representing 45% of the national production. In Madrid there is only one autochthonous goat breed, Cabra del Guadarrama, in danger of extinction. Its name comes from the Sierra de Guadarrama, a mountainous region located in Madrid and Castilla y León, where the main group of animals of this racial entity is exploited, so there are farms of this native breed in the Spanish Autonomous Communities of Madrid and also in Castilla-León (mainly in Ávila and Segovia).

The farms are mainly used for meat production, although they are also used secondarily for milk production. The milk is largely used for the production of cheese, normally being mixed with the milk of other more productive breeds. Goat milk has some special and unique aroma and flavor characteristics as well as nutritional and health values [4]. Milk composition can be influenced by many factors, including breed, genetics, physiology, feed, environment and others [5]. The physico-chemical composition, microbiological and sensory properties of cheese depend on many factors such as milk composition, production technology (industrial or artisanal), curd cheese (composition and handling), salting technology and ripening period, among others, which influence the final quality of cheeses [6]. Several studies have been conducted around the world on the composition and quality of milk, which are influenced by various factors. There is not enough information from local studies on the nutritional composition of milk produced at the farm level [7].

Interest in goat dairy products is increasing due to their functional properties and high nutritional values [8]. The production of different Spanish cheeses, elaborated with milk from autochthonous breed, represents an important economic income and promotes the protection of indigenous genetic resources, characterized by a strong and specific relationship with the territory. Furthermore, in recent years, there has been an increase in the number of artisan cheese dairies producing sheep and goat cheese in Madrid [9], although there are very few cheeses that are made only with milk from the native breed of Madrid, the Cabra del Guadarrama goat [10].

There are no recent studies on the physico-chemical, microbiological and sensory characteristics of cheeses from the Madrid autochthonous Cabra del Guadarrama goat breed, nor any comparing them with those of other goat breeds.

Moreover, the characterization of different cheeses may contribute to the valorization of the autochthonous breeds and increase their consumption. Therefore, the aim of the present study was to describe four goat cheeses produced in artisan cheese dairies with milk from different autochthonous goat breeds in order to determine their physico-chemical composition, microbiological and sensory characteristics and acceptance by consumers, in order to contribute to the preservation of autochthonous breeds. In addition, we evaluated the effect of the information that the cheeses were made with milk from native breeds (one of them from Madrid) had on consumer acceptance.

## 2. Materials and Methods

### 2.1. Material: Goat Cheeses

Samples of four cheeses produced with raw goat milk from different autochthonous Spanish breeds were studied in order to compare their physico-chemical, microbiological and sensorial characteristics. The hard-pressed paste cheeses were made by four different artisanal cheese factories and ripened for 60 days. The cheese samples were as follows: (1) hard goat cheese from the Guadarrama goat breed (CGC), an autochthonous breed of Madrid produced by Quesería Montealijar (Navas del Marqués, Ávila); (2) hard goat cheese from the Malagueña breed (MC) made in the cheese factory La Caperuza (Bustarviejo, Madrid); (3) hard goat cheese from the Murciano-Granadina breed (MGC), a native breed of Murcia, made in the farm cheese factory Puerto Carrillo (Benaocaz, Cádiz) and (4) hard goat cheese from the Florida breed (FC), a native breed of Sierra Morena, made by the Corsevilla cheese factory (Cazalla de la Sierra, Sevilla). All the cheeses were produced in March 2023, and the ripening period was completed in May 2023. After the end of the ripening period, they were transferred to Instituto Madrileño de Investigación y Desarrollo Rural, Agrario y Alimentario (IMIDRA) and stored at 4 °C until processing. For each type of cheese, two independent samples were analyzed. For the analysis, two different cheeses elaborated in two independent trials were taken for each cheese (two cheeses samples for elaboration, four cheeses for cheese factory in total). All assays were performed in triplicate.

#### 2.1.1. Characteristics of the Livestock of Each Dairy

The Montealijar Guadarrama bree dhas 105 Guadarrama goats in an extensive system, as the grazing of the herd varies according to the time of year, adapting to the changes in the vegetation of the mountains. The herds mainly eat rockroses, grasses, brambles, juniper, pine forest soil and thyme, and are fed with cereal mix, maize, oats, pea and sunflower seeds. Evolutionary trend: recession.

The La Caperuza Malagueña breed has 170 goats in an extensive system. The goats graze daily in the valley of Bustarviejo for between 7 and 8 h a day, on a farm of more than 200 ha of oak and scrubland. The variety in their diet means that they have changing organoleptic conditions. Evolutionary trend: recession.

The Puerto Carrillo Murciano-Granadina breed has 150 goats in an extensive system, feeding on plants (mastic, wild olive, pastures of Sierra de Grazalema) and unifeed feed for dairy goats. Evolutionary trend: recession.

The Corsevilla Florida breed is reared in the pasture, where they graze freely in an extensive system. Evolutionary trend: expansion.

#### 2.1.2. Cheese Manufacturing

The production of all cheeses was carried out at the same time in each of the different dairies, using a comparable production process. First, the milk was heated to between 31 and 32 °C. Then, starter cultures (the indigenous microorganisms in MGC) and calcium chloride were added until the pH was 6.4. The raw goat milk was coagulated with kid’s rennet for one hour and then cut to the size of a rice-lentil bean. The temperature was then gradually increased to 37 °C. The whey was separated from the curd. The curd was molded and then subjected to a series of 30 min cycles of pressure, with the pressure gradually increasing to 1.5 kg. The cheeses were then salted in CGC, MC and FC by placing them in immersed brine (17% salt) and placing MGC in dry brine. Finally, the ripening time was 60 days at 7–12 °C and 85–95% humidity. At the end of the ripening time, the approximate weight of the cheeses was 0.8 kg for FC, 1 kg for CGC and MC and 2 kg for MGC. Finally, the cheeses were vacuum-packed, transferred to IMIDRA and stored at 4 °C until analysis, which was carried out less than two weeks after receipt. Table 1 describes the characteristics of the livestock and the cheeses.

Samples of four cheeses produced with raw goat milk from different autochthonous Spanish breeds were studied in order to compare their physico-chemical, microbiological and sensorial characteristics.

### 2.2. Composition and Physico-Chemical Analyses of Cheeses

The composition analysis and physico-chemical analysis of the goat cheeses included fat, proteins, salt content, pH, moisture and water activity (a_w_). Total lipid content was determined by the Soxhlet method [11]. Total protein content was determined by the Kjeldahl method (total nitrogen was measured by the Kjeldahl method and factor 6.25 was used for the conversion of nitrogen to crude protein) [12]. Both fat and protein content analysis were performed by the Laboratorio Regional de Sanidad Animal de la Comunidad de Madrid (Madrid). Salt content was determined by chloride analysis and was carried out using the photometric analyzer CDR FOODLAB^®^ (CDR s.r.l, Florence, Italy) based on the reaction of chloride ions with mercuric thiocyanate. Thiocyanate was released, giving rise to an orange complex, whose intensity, measured at 505 nm, was directly proportional to the concentration of chlorides in the sample. For this purpose, 2 g of cheese sample was homogenized with a 0.25M NaOH solution. Then, according to the manufacturer’s instructions, the sample was placed in the kit and all measurements were performed in triplicate. The results were expressed as g NaCl/100 g cheese. A pH meter (Hanna Instruments HI5521, Lingolsheim, France) was used to measure the pH values; moisture content was determined as described in AOAC-925.10 [13]. The a_w_ of cheeses was detected by using the LabMASTER-a_w_ (Novasina AG, Lachen, Switzerland), which was periodically calibrated.

### 2.3. Fatty Acid Profile of Cheeses

To determine the fatty acid profile of the cheese samples, they were previously lyophilized and later derivatized with sodium methoxide 0.5 M in methanol and acetyl chloride in methanol (1:10 *v*/*v*), with tridecylic acid (C13:0) as an internal standard, by gas chromatography [14]. Fatty acid methyl esters (FAMEs) were extracted with hexane. A gas chromatograph (Agilent 7820A, Agilent Technologies, Santa Clara, CA, USA) fitted with an Agilent DB-23 capillary column (60 m × 0.25 mm i.d., 0.2 μm) was used with a flame ionization detector. The injector and detector temperatures were 250 and 260 °C, respectively. Helium was used as a carrier gas at 1 mL/min. The sample injections (1 μL) were performed in a split ratio: 80:1. The oven temperature at 50 °C was held for 1 min and increased at a rate of 25 °C/min to 175 °C. It was then increased at a rate of 4 °C/min to 230 °C, being held for 16 min. The quantitation of FAMEs was performed by relative response factors with respect to the internal standard of tridecylic acid. The analyses were performed in triplicate. This quantification was performed by the Analysis Service Unit facilities of the Institute of Food Science, Technology and Nutrition (ICTAN-CSIC, Madrid).

Health-related lipid indices of the processed cheeses were assessed using different combinations and ratios between fatty acids, such as all short-chain fatty acids (ΣSCFA), total saturated fatty acids (ΣSUFA), total monounsaturated fatty acids (ΣMUFA), total polyunsaturated fatty acids (ΣPUFA) and all unsaturated fatty acids (UFA = ΣMUFA + ΣPUFA). The following lipid quality indices were calculated:

- Index of atherogenicity (AI) = $\frac{C12:0+(4\;\times \;C14:0)+C16:0)}{\Sigma \text{-}3\mathrm{PUFA}+\Sigma \text{-}6\mathrm{PUFA}+\Sigma \text{-}\mathrm{MUFA}}$;
- Index of thrombogenicity (TI):
  $$\frac{(C14:0+C16:0+C18:0)/(0.5\;\times \;C18:1)+(0.5\;\times \;\mathrm{other}\;\mathrm{MUFA})+(0.5\;\times \;\Sigma \text{-}6\mathrm{PUFA})+(3\;\times \;\Sigma \text{-}3\mathrm{MUFA})}{(\Sigma \text{-}3\mathrm{PUFA}/\Sigma \text{-}6\mathrm{MUFA})};$$
- Hypocholesterolemic fatty acids (DFAs) = UFA + C18:0;
- Hypercholesterolemic fatty acids (OFAs) = Σ SUFA − C18:0;
- Hypocholesterolemic and hypercholesterolemic FA ratio (H/H) = DFA/OFA;
- Ratio = ∑n-6/∑n-3.

The lipid quality indices were calculated as described by Paszczyk et al. [15].

### 2.4. Color and Texture Profile Analysis

Cheese color was evaluated using the photoelectric tristimulus colorimeter (CHROMAMETER CR-200, Konica Minolta, Tokyo, Japan) using the D65 illuminant. Three color parameters were determined for all samples: L* (lightness), a* (green–red) value and b* (blue–yellow) value. Color measurements were determined in both the paste (interior) and the rind, and were measured according to the CIELab color space system [16].

The texture profile analysis of the cheese was carried out in a TA-XT2 texturometer using cylindrical samples of 1 cm in height from each cheese sample (8 cylindrical representative pieces were obtained). The samples were compressed twice at 50% and 75% height with a cylindrical aluminum probe 35 mm in diameter (P35) at 2 mm/s with a 10 s delay between compressions. From the force vs. time texturograms, six parameters were obtained for compression: hardness (N), springiness, cohesiveness, adhesiveness (Ns), chewiness (N), resilience and gumminess (N). The mechanical parameters were calculated from the force–displacement curve obtained [17].

### 2.5. Microbiological Analysis

Ten grams of cheese were aseptically collected and transferred into a bag, diluted ten-fold in sterile peptone water and homogenized using a Stomacher 400 (Seward Laboratory, London, UK) for 180 s. Serial dilutions of the sample homogenate were prepared in 0.1% sterile peptone water and microbial counts were determined on duplicate plates of the corresponding medium. Decimal dilutions of the cheese samples were plated. The following microbial groups were assessed: (a) lactobacilli incubated anaerobically on Man Rogosa Sharpe (MRS) agar at 37 °C for 72 h; (b) lactococci on M17 agar at 37 °C for 72 h; (c) heterofermentative lactic bacteria (HLB) on APT agar at 37 °C for 72 h; (d) mesophilic aerobic bacteria (MAB) on plate count milk agar (PCA) at 30 °C for 72 h; (e) mold and yeast on potato agar dextrose (PDA) at 30 °C for 72 h; and (f) Enterobacteriaceae on violet red bile glucose agar (VRBG) after incubation at 37 °C for 24 h. Other genera of lactic acid bacteria, such as *Pediococcus* and *Leuconostoc,* were also grown in MRS medium. Small, opaque and white colonies were taken into account for counting, while colonies without these characteristics were excluded. All assays were performed in sterile conditions and were carried out in triplicate. The results were expressed as log CFU (Colony Forming Units)/g cheese.

### 2.6. Sensory Analysis

#### 2.6.1. Quantitative Descriptive Analysis (QDA)

A quantitative descriptive analysis (QDA) was performed by a panel of 14 trained assessors (seven females and seven males) at IMIDRA who were experts in the characterization of dairy products, and who received training in the use of scales and in the evaluation of the different sensory attributes tested, as well as familiarization with the standard food scales used [18]. The judges evaluated the attributes of appearance, texture, flavor profile and olfactory profile of the cheeses through the visual phase, tactile phase, olfactory–taste phase and flavor (or mouth) phase. The sensory evaluation of the cheeses was carried out at 16 °C. Samples, coded with random 3-digit codes, were presented in a balanced way to avoid the effect of the presentation order. Cheeses were served without any identification of the origin of the milk used (Cabra del Guadarrama, Malagueña, Murciano-Granadina and Florida). Panelists were provided with room-temperature water and unsalted breadsticks between samples. The samples were prepared to be equal in size and shape (1 × 1 cm cubes). The sensory analysis was carried out in the tasting room of Finca El Encín, IMIDRA (Alcalá de Henares, Spain), equipped with an environment of constant and uniform illumination with natural light, isolated from noise, free of foreign smells, with good ventilation and with comfortable temperature and humidity conditions. The samples were prepared in a separate room from the tasting room. The samples were rated using interval linear graphical scales 10 cm long, scoring 1 (lowest)–10 (highest), and the average of the panelists’ scores was calculated. The linear graphic scale provided continuous data limited by the precision of the measuring, which approximated a normal distribution and generated continuous data.

The panel assessed each particular element of quality such as springiness (scale: nil to high; foods: butter, stuffed olive and sausage), surface roughness (scale: smooth to sandy; foods: apple and different biscuit cuts), surface humidity (scale: dry to wet; foods: walnut shell, orange rind, inside of banana peel and cut apple), firmness (scale: nil to high; foods: spreadable cheese, sausage and cooked carrot), friability (scale: nil to high; foods: boiled egg white, muffin and wafer), adherence (scale: nil to high; foods: boiled egg white, boiled egg yolk and toffee caramel), juiciness (scale: dry to juicy; foods: biscotte, banana, apple, orange and watermelon), number of chews, flavors (sour, salty, bitter, sweet), identification and intensity of different aromas perceived directly (through the nose) and retronasally, and finally, persistence and overall impression [18]. For persistence the lowest third of the scale (0–3.33 points) were used if the sensation persisted for 5 s or less; the middle third (3.34–6.66 points) if it persisted for between 5 and 10 s; and the highest third (6.67–20 points) if it persisted for more than 10 s.

#### 2.6.2. Consumer Acceptance Test

Consumer acceptance testing was conducted to determine consumer preferences for the flavor and texture of goat cheeses made from the milk of different autochthonous goat breeds (Cabra del Guadarrama, Malagueña, Murciano-Granadina and Florida). Consumers (n = 116, 61 females and 55 males, age range from 18 to 74 years) were recruited at the IMIDRA (Madrid, Spain). The participation of the consumers was voluntary and no monetary compensation was given. To be eligible for participation, consumers had to be regular consumers of goat cheeses (at least twice a month) and be interested in and available to participate in the study. Sensory evaluation was performed in different sessions. The survey was carried out using the SENSESBIT online software version 4.5.1 (Tastelab, Lugo, Spain) via mobile phone with access via QR code. Cheese samples were offered at 15 °C and coded with a three-digit number. Samples were served in blind and informed conditions and in a completely randomized order. Consumers conducted a hedonic test to rate the aspects, flavor, smell, texture and their overall liking of the four types of cheese. A 9-point Likert scale was used from 1 to 9 (from dislike extremely (1) to like extremely (9)) for the sensory evaluation. Consumers were asked about their purchase intention regarding the cheeses studied by means of a five-point scale with the terms “certainly not”, “probably not”, “maybe yes/maybe no”, “probably yes” and “definitely yes”, and finally, an ordering test was carried out for these goat cheeses. In the rank order test, the panelists ranked samples according to their order of preference. A 4-point scale (1 corresponded to the lowest preference and 4 to the highest preference) was used. Preference was obtained by calculating the sum of the products of the values given to each sample (from 1 to 4) by the number of times that each sample was allocated to a specific score.

### 2.7. Statistical Analysis

Statistical analyses of the composition and physico-chemical and microbiological characteristics; quantitative descriptive analysis; and the hedonic results of goat cheeses were performed using a one-way ANOVA with Tukey’s test for assessing the differences between samples. Furthermore, Pearson’s correlation analysis was used to analyze the relationship between the composition; physico-chemical and microbiological characteristics; quantitative descriptive analysis; and hedonic sensory results from the cheese samples. A statistical analysis of rank order test data was determined by means of Friedman’s rank test. Principal component analysis was performed by means of the varimax method, an orthogonal rotation method that minimizes the number of variables that have high loadings on each factor and simplifies the interpretation of the factors. The profiles (fatty acid percentages) of the cheeses were classified using a hierarchical cluster analysis. Differences were considered significant at *p* ≤ 0.05. The data of ordinal variables such as purchase intention were analyzed using contingency tables and a Chi-square test. All calculations were conducted in the SPSS 25.0 statistical package (SPSS Inc., Chicago, IL, USA).

## 3. Results and Discussion

### 3.1. Composition and Physico-Chemical Analyses of Cheeses

The physico-chemical composition of goat cheeses (fat, proteins and salt content) is presented in Table 2. The fat content ranged from 31.28 to 39.26%, and all cheeses could be classified as semi-fat cheeses according Spanish legislation [19]. The results showed a lower fat value in CGC, then in FC, MC and finally in MGC. Significant differences were found between all cheeses (*p* < 0.05). The fat content values found in our study for cheese made from Murciano-Granadina and Malagueña goat milk were higher than reported for these cheeses in the literature [20,21]. Many of the variations in cheese characteristics were due to the differences in milk composition that depend on lactation stage, season, breed, feeding and genotype [5]. Furthermore, when the milk is not standardized, as in artisanal cheeses, the effect of the breed and the ripening time are the most influential factors on the physico-chemical characteristics of cheeses [3,20].

The protein content ranged from 22.34 to 26.23% and statistically significant differences (*p* ≤ 0.05) were observed. CGC and MG (26.20 and 26.23%, respectively) had a higher protein content than MGC (23.45%) and FC that showed the lowest fat percentage (22.34%). The fat/protein ratio of goat milk normally varies between 1.1 and 1.5 [5]. The variation in the fat/protein ratio between cheeses made from the milk of different indigenous goat breeds ranged from 1.19 to 1.67, with significant differences (*p* < 0.05). All cheeses were made in spring, so that could have presented a high fat/protein ratio which could have led to a reduction in the fat retention in the cheese during its transformation [22].

According to the data from the milk control, the milk of the Malagueña breed goat has, on average, 4.8% fat and 3.4% protein. Similarly, the milk of the Guadarrama goat breed has 4.6 and 3.5% fat and protein, respectively; milk of the Florida breed has 4.9% fat and 3.4% protein; and finally, milk of the Murciano-Granadina breed has 5.1% and 3.6% fat and protein [23], although, of course, these are average values that change depending on the state of lactation, the time of year or the feeding of the animals. The composition of the cheeses studied (percentage of fat and protein) is generally correlated with the composition of the milk used to make the cheeses, although these data are not available in this study.

The four cheeses had less than 2% of salt. The CGC salt content was lower than the rest of the cheeses studied (1.25% salt) (*p* < 0.05). There were no significant differences between MC, MGC and FC (*p* < 0.05), obtaining values between 1.72 and 1.87%. NaCl content was similar with that found in the literature for hard cheeses [24]. The differences between the cheeses studied could be due to variations in the specific salting process used for each particular cheese. It is possible that more salt is added during the salting process, resulting in a higher salt content in the final product. The addition of salt in high concentrations to increase the flavor and preservation of a cheese decreases proteolytic enzyme activity while increasing osmotic pressure, which eliminates part of the water trapped in the protein network of the curd, causing cheeses to have lower moisture [25]. Also, salt dehydrates bacteria, killing them or preventing them from growing and multiplying [6]. Namely, a high correlation coefficient was observed between fat and salt content (r = 0.931, *p* > 0.05) (Table S2).

The physico-chemical parameters of pH, moisture and water activity (a_w_) can be seen in Table 1. In relation to pH, the values obtained are in the range between 4.85 and 5.39. The pH of the cheeses should be slightly acidic to inhibit the growth of pathogenic microorganisms. MGC was the most acidic cheese (pH = 4.85), followed by FC (5.27). CGC and MC were lower in acidity (5.36 and 5.39, respectively) than MGC and FC. Significant differences (*p* < 0.05) were observed between MGC and FC, and between each of them and CGC and MC. Low pH values (particularly below 5.0) in artisanal cheeses could be a relevant factor for pathogen control [26]. In general, pH measurement is an important tool to verify appropriate fermentation; the pH of hard cheeses tends to increase with the ripening process due to the presence of certain basic amino acids and NH_3_, as well as the decomposition of the lactic acid salt [27,28]. Furthermore, the type of starter culture used, the initial microbial load of the milk used and the absorption of salt during the salting process could have contributed to the observed variations in pH [21].

Moisture content in cheeses from milk of different autochthonous goat breeds showed significant differences (*p* < 0.05). CGC and FC have a higher moisture content (37.48 and 36.30%, respectively) than MGC and MC (33.01 and 30.77%, respectively). Considering that the ripening time of the cheeses was 60 days, it could be expected that they had low moisture values. The moisture of most cheeses is established by the speed and extension of syneresis and the compression of the structure of casein. After coagulation, processes such as molding, pressing and salting are coupled with a lowering of pH and result in a significant loss of moisture as the whey is removed [3].

Related to water activity, significant differences (*p* < 0.05) were observed among the different cheeses studied. Values of a_w_ ranged between 0.93 and 0.95 and statistically significant differences were detected between all cheeses. CGC and MC had lower a_w_ than FC and MGC. Moisture in cheeses was related to a_w_. In this sense, CGC was found to have the highest moisture content among the cheeses analyzed, indicating a higher a_w_ compared to the other cheeses. In second place was FC, followed by MC and MGC, which presented similar moisture values and a lower a_w_ compared to the two previous cheeses. To corroborate these relations, a significant correlation coefficient was observed between a_w_ and moisture (r = 0.983, *p* < 0.05) (Tables S1, S2 and S4).

The cheeses made from the milk of different autochthonous goat breeds studied were different in fat, protein, salt, pH, moisture content and a_w_,which could be due to the different compositional characteristics of the milk that was used for their manufacture, and also to the different manufacturing procedures, even though they were all manufactured from raw milk collected in the same month and were ripened for 60 days.

### 3.2. Fatty Acid Profile of Cheeses

The fatty acid (FFA) compositions of cheeses made from the milk of different autochthonous goat breeds (Cabra del Guadarrama, Malagueña, Murciano-Granadina and Florida) are presented in Table 3. The fatty acid profile is described by 26 fatty acids. The following are the most predominant fatty acids that were identified in cheeses: palmitic acid (C16:0), oleic acid (C18:1n9c), stearic acid (C18:0), myristic acid (C14:0), capric acid (C10:0), lauric acid (C12:0), caprylic acid (C8:0) and linoleic acid (C18:2w-6). In MGC, palmitic acid (C16:0), capric acid (C10:0), lauric acid (C12:0) and myristic acid (C14:0) content were higher than in CGC, MC and FC (*p* < 0.05). CGC had lower oleic acid (C18:1n9c) and stearic acid (C18:0) content than rest of cheeses (*p* < 0.05). Only in MGC was EPA-Timnodonic acid (C20:5 n3) detected. The results obtained showed that saturated fatty acids (SFAs) were the predominant fatty acids of all the cheeses studied. CGC had a significantly lower (*p* < 0.05) content of these acids (162.73 ± 4.26 mg FAME/g cheese) than MGC (212.03 ± 2.69 mg FAME/g cheese), which were found to be predominantly caproic acid (C6:0), caprylic acid (C8:0), capric acid (C10:0), lauric acid (C12:0) and palmitic acid (C16:0). SUFAs are known to contribute to the characteristic flavor and aroma of cheese [29]. Among the MUFAs detected in these cheeses, the most abundant was oleic acid and no significant differences were detected between MC, MGC and FC (*p* > 0.05), while CGC had a lower content of this fatty acid than the rest of the cheeses. The UFA contents were lower in CGC (56.00 ± 1.38 mg FAME/g cheese), with significant differences with the other autochthonous goat cheeses in our study. CGC had a lower content of all fatty acid fractions, due to the lower fat content than the rest of the cheeses studied. The flavor properties of cheese are directly influenced by the amount of FFAs and the pH value, and these parameters tend to influence each other [30]. In our study, a negative correlation was found between pH and SCFA and SUFA content (r = −0.978 and r = −0.894, respectively, *p* < 0.05) (Table S2).

To evaluate the nutritional value of the lipids of the cheeses, different lipid health indices were calculated. The PUFA n-6/n-3 fatty acid ratio is an important health factor and maintaining a low omega-6/omega-3 ratio is important for decreasing inflammation [31]. The Western diet, with its excessive consumption of processed foods and inadequate intake of omega-3 fatty acids, is characterized by a high PUFA n-6/n-3 ratio of up to 15/1-16.7/1 [32]. In our study, there were significant differences between CGC and MC that showed a significantly lower (*p* < 0.05) n-6/n-3 ratio (3.73 and 3.76, respectively) than FC (4.85) and much less than MGC (11.56). Furthermore, higher values were obtained (the observed ratio was in the range of 3.74–11.00) than those found in the literature for semi-hard cheeses, where the ratio was 3.3 [33]. Another study found that the PUFA n6/n3 ratio was 2 for cheeses made from Carpathian goat milk [34]. CGC and MC, which showed a lower n-6/n-3 ratio, are cheeses made in Madrid and Las Navas del Marqués, a town near to the Madrid region. CGC is made using milk from the autochthonous Cabra del Guadarrama breed. Although there may be differences due to breed, animal feeding is perhaps the most important factor influencing the w6/w3 ratio. Feeding systems, particularly grazing, have been shown to affect the profile of essential fatty acids, particularly the omega-6/omega-3 balance. The utilization of concentrates probably increases the omega-6 content or decreases the omega-3 concentration, often exceeding a 4:1 omega-6/omega-3 ratio and diminishing the beneficial effects of omega-3 on consumer health [35]. Conversely, goats that graze on natural pastures tend to produce milk and cheese with a more favorable omega-6/omega-3 ratio. This is because pastures are richer in omega-3 fatty acids. Therefore, the difference is probably due to the type of feed used, as well as the higher proportion of concentrates [36]. Another study found that the content of fatty acids varied according to breed, with significant differences evident not only for individual acids, but also for their groups, such as SFAs, UFAs and MUFAs [37].

A useful parameter that indicates the health properties of food is the AI (showing the inhibition of the aggregation of plaque), and it is assumed that the most favorable AI value for human health is below 1 [38]. It was observed that MGC had a higher AI than the rest of the cheeses studied. Another interesting index is the thrombogenic index (showing the tendency to form clots in the blood vessels). In the present study, the lowest atherogenic and thrombogenic index was determined in cheeses made from the milk of the Malagueña goat breed (2.65 and 3.35, respectively), while the highest value was recorded in cheese made from the milk of Murciano-Granadina goats (3.03 and 4.08, respectively), with significant differences (*p* < 0.05). When we compared our results, we found that lower values of AI were obtained for cheeses made with milk from different Polish goat breeds [38], but that these values were similar to those obtained for cheeses made with milk from Bulgarian goat breeds [39]. In addition, cheese made of milk from the autochthonous del Guadarrama breed was characterized by the lowest hypocholesterolemic and hypercholesterolemic fatty acid index (84.68 and 92.97, respectively) of all the cheeses studied (*p* < 0.05). Therefore, the influence of breed on the fatty acid profile was visible in our study, similar to that described in the literature [39]. Moreover different authors have described that the feeding system plays a major role in modulating the fatty acid composition of cow, goat and sheep milk [40,41,42,43] and have also described an improvement in the nutritional quality of cheeses produced in the spring months [22].

The four cheeses studied have different fat contents, so the fatty acid content depends on the amount of total fat and therefore on the total fatty acid content. The percentage of each fatty acid in relation to the total amount of fatty acids has also been calculated. This is because the percentage of fatty acids is a better indicator of the quality of the fat in cheeses. The results are expressed in Table 4. It can be seen that no statistically significant differences were observed for the butyric acid (C4:0) and myristic acid (C14:0) fatty acid percentages among all cheese types studied. However, the four cheeses were all different in the percentage of the following fatty acids: palmitic acid (C16:0), margaric acid (C17:0), linoleic acid (C18:2n6c), arachidic acid (C20:0), C20:1n9 and behenic acid (C22:0). MGC had the highest percentage of palmitic acid (C16:0) fatty acid (30.21%), then FC (28.67%), CGC (27.53%) and MC (26.74%). The percentage of the fatty acid margaric acid (C17:0) varied from 0.47% (MGC) to 0.58% (FC), 0.71% (CGC) and 0.96% (MC). They were also all different in the percentage of linoleic fatty acid (C18:2n6c). The one with the lowest percentage was CGC (2.22%), followed by FC (2.76%), MC (3.28%) and MGC (3.67%). In relation to the percentage of the fatty acid arachidic acid (C20:0), MGC had 0.20%, FC 0.41%, CGC 0.70% and MC 0.96%. CGC had a lower percentage of C20:1n9 (0.05%) than FC (0.07%), MGC (0.07%) and MC (0.09%). In the same way, MGC had a lower percentage of the behenic acid (C22:0) fatty acid (0.06%) than FC (0.12%), CGC (0.21%) and MC (0.29%).

**Figure 1:**
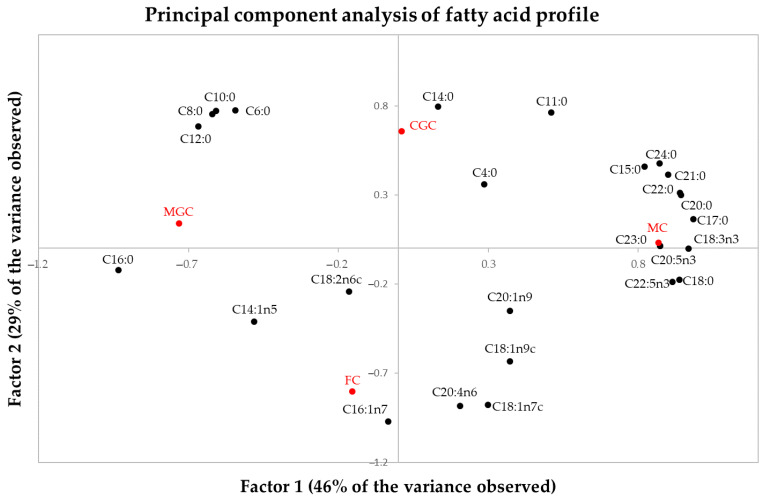
Distribution of the percentages of fatty acids in the map configured by the first two principal components. CGC = Cabra del Guadarrama Cheese; MC = Malagueña Cheese; MGC = Murciano-Granadina Cheese; FC = Florida Cheese. The position of the studied cheeses is shown in red lettering.

Figure 1 shows the distribution of the percentages of fatty acids in the map configured by the first two principal components, which together explain 76% of the observed variance.

MC had a higher percentage of the margaric acid (C17:0), stearic acid (C18:0), α-linolenic acid (C18:3n3), arachidic acid (C20:0), timnodonic acid-EPA (C20:5n3), C20:1n9, C21:0, behenic acid (C22:0), clupanodonic acid-DPA (C22:5n3), C23:0 and lignoceric acid (C24:0) fatty acids and a lower percentage of the myristoleic acid (C14:1n5) and palmitic acid (C16:0) fatty acids than all other cheeses studied. MGC had a higher percentage of palmitic acid (C16:0) and linoleic acid (C18:2n6) and a lower percentage of the pentadecylic acid (C15:0), margaric acid (C17:0), α-linolenic acid (C18:3n3), arachidic acid (C20:0), behenic acid (C22:0) and clupanodonic acid-DPA (C22:5n3) fatty acids than all other cheeses studied. FC had a higher percentage of the myristoleic acid (C14:1n5), palmitoleic acid (C16:1n7) and arachidonic acid (C20:4n6) fatty acids than the rest of cheeses studied. CGC had the lowest percentage of the palmitoleic acid (C16:1n7), cis-vaccenic acid (C18:1n7c), linoleic acid (C18:2n6c) and C20:1n9 fatty acids than the rest of the cheeses studied.

MGC and CGC had a higher percentage of short- and even-chain saturated fatty acids (caproic acid (C6:0), caprylic acid (C8:0), capric acid (C10:0) and lauric acid (C12:0)) and a lower percentage of the cis-vaccenic acid (C18:1n7c) and arachidonic acid (C20:4n6) fatty acids than MC and FC.

CGC and MC had a higher percentage of odd-chain saturated fatty acids: hendecanoic acid (C11:0), pentadecylic acid (C15:0), margaric acid (C17:0), (C21:0) and long-chain even-chain saturated fatty acids (arachidic acid (C20:0), behenic acid (C22:0), lignoceric acid (C24:0)) and a lower percentage of palmitic acid (C16:0) than FC and MGC.

**Figure 2:**
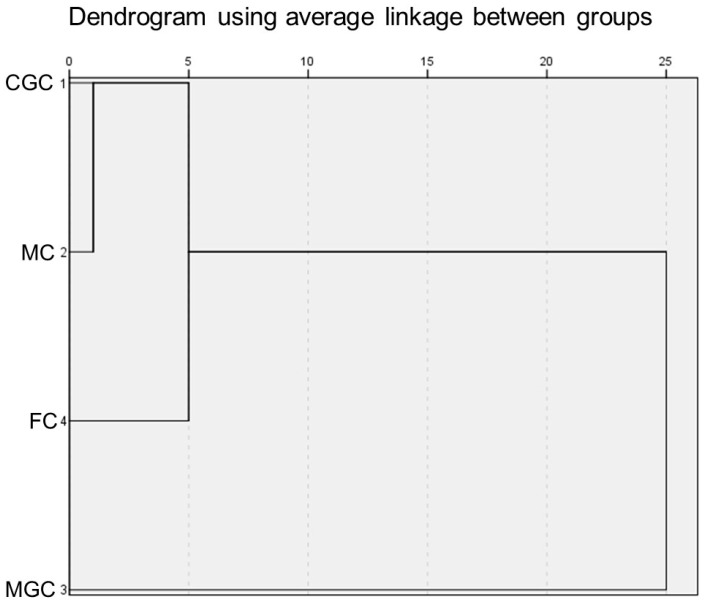
Dendrogram of goat cheeses made from the milk of different autochthonous goat breeds. CGC = Cabra del Guadarrama Cheese; MC = Malagueña Cheese; MGC = Murciano-Granadina Cheese; FC = Florida Cheese.

A dendrogram was constructed using the percentages of fatty acids in the studied cheeses as variables. The results showed that the fatty acid profile of Guadarrama Goat cheese was more similar to Malagueña Cheese than to the Florida and Murciano-Granadina goat cheeses (Figure 2). These two cheeses were produced in the Madrid and Ávila regions of Spain, less than 90 km apart. In contrast, the Florida and Murciano-Granadina Cheeses were produced in Andalusia, in the provinces of Seville and Cádiz, respectively. Murciano-Granadina goat cheese is the one with the most different fatty acid profile compared to the rest of the cheeses studied. As previously discussed, MGC had a higher atherogenic and thrombogenic index, and a higher PUFA n-6/n-3 ratio than the rest of the cheeses studied. In Spain, several breeds of goat are highly prized for the quality of their milk, which has been used for cheese production. The Murciano-Granadina breed is the most widely used for this purpose. The Murciano-Granadina breed has high milk production and is highly specialized in this respect, surpassing others in volume. Its milk has a high fat and protein content, which translates into an excellent cheese yield and a characteristic flavor. In addition, MGC has a higher fat content than the other cheeses. The Murciano-Granadina breed is the most widely used in Spain for producing goat cheese thanks to its high milk production. This breed enables the production of cheeses with a good yield and high sensory quality, although the lipid profile of its milk is less healthy than that of other breeds used by small-scale dairies to make artisanal cheeses.

### 3.3. Color and Texture Profile Analysis of Cheeses

The color of the rind and the paste (interior) of the cheeses made from the milk of different autochthonous goat breeds are presented in Table 5. The colorimetric parameters of cheese depend mainly on the type of milk used in the production of the product and the ripening time. When the color of the cheese paste was analyzed, the lightness (L*) of the cheese paste ranged from 77.34 for MC to 89.98 for MGC and it was found that CGC and MC had lower luminosity values than the other two cheeses (81.34 and 77.34) (*p* < 0.05). No differences were observed in the red index among the four cheeses studied.

Similar values were found in a study of Canarian goat cheeses [3]. They described that goat cheese was the brightest in comparison to cheese from other animal species. In the red index of cheese paste (a*), no significant differences (*p* > 0.05) were found among the cheeses studied. Finally, in the yellow index of cheese paste (b*), significant differences (*p* < 0.05) were observed among the four types of cheese studied.

Significant differences in rind lightness (L*) (*p* < 0.05) among the four cheeses studied were measured, with a lower brightness in CGC and higher brightness in FC (55.80 and 70.99, respectively), although no significant differences (*p* > 0.05) were found between MC and MGC.

Statistically significant differences were observed among the four cheeses analyzed for the colorimeter parameters of the rind red (a*) and yellow (b*) index (*p* < 0.05). The highest redness and yellowness values were found for MGC, followed by MC, CGC and FC.

The color of cheeses is influenced by the type of milk used, by the processing technique or family to which it belongs and by the ripening time. Also, the feeding system given to goats has a significant impact on the levels of carotenoids in their milk and the color of the cheese produced [44]. In the literature it has been described that cheeses made with spring milk are more yellowish in color than those made with winter milk. For example, cheeses made with winter milk from dairy goats fed grass silage are more yellowish than those made with milk from animals fed hay. On the other hand, it has been described that ripening time affects all color parameters in most cheeses [3]. The high L* value of cheese could be due to the high moisture content [45]. The redness (a*) observed as ripening progressed may have been related to the chemical composition (smaller fat globules and a total conversion of β-carotene to vitamin A). The yellow color (b*) of the cheese was mainly due to the presence of carotenoids [46]. The increases in the a* and b* values were due to biochemical reactions, such as lipolysis and proteolysis during maturation and the diversified feeding of the goats [47]. Some researchers related increases in the L* and b* of the cheese surface to microstructural changes [48].

To study the texture of the goat cheeses, a double compression test (TPA) (at both 50% and 75% of the initial size) was carried out. Table 5 shows the results obtained for the instrumental texture properties of goat cheeses. At 50% compression, it was observed that CGC (46.07 N) and FC (53.91 N) were significantly softer (*p* > 0.05) than MC (76.06 N) and MGC (70.86 N) and there were no significant differences (*p* > 0.05) between CGC and FC and between MGC and MC. The most elastic cheese was CGC (0.87), and the least elastic was FC (0.76), although there were no significant differences (*p* > 0.05) between CGC, MC and MGC. Cabra del Guadarrama Cheese is also more cohesive (0.53) (*p* > 0.05) than the other cheeses studied. CGC and FC (0.28 and 0.20 Ns, respectively) were statistically significantly more adhesive cheeses (*p* < 0.05) than MGC and MC (0.06 and 0.02 Ns, respectively). Higher values of hardness and lower values of cohesiveness than the cheeses studied in this work have been described for Canarian goat cheeses also ripened for 60 days [3]. FC had lower values of chewiness (16.45 N) than MC (23.30). However, no significantly differences were detected among MGC, CGC and FC (*p* > 0.05). CGC had a higher value of resilience (0.33) than the rest of the cheeses in this study (*p* < 0.05). Finally, there was a significant difference (*p* < 0.05) between MC and FC with regard to gumminess, which was higher for MC (29.56 N) than for the other cheeses. Similar values have been described for the springiness, resilience and cohesiveness of Mexican cheeses [26].

When the test was carried out with a compression of 75%, all the values obtained followed the same trend as those obtained with 50%, with the exception of adhesion, which was higher for FC (0.18 Ns), and chewiness, which was higher for MGC (11.96 N).

The results of the instrumental texture analysis performed showed that MGC and MC are harder cheeses than CGC and FC. The texture properties of these two cheeses are more similar to each other than to those of MC and MGC.

Textural characteristics are criteria of great importance to the sensory properties and the perception and acceptance of products by consumers, so several studies have reported correlations between chemical composition and selected instrumental textural parameters of cheese [49]. For example, protein content in cheese was positively associated with firmness and chewiness but negatively associated with gumminess [50]. In contrast, in this study, protein was positively associated with gumminess at 75% compression (r = 0.930). Fat content was reported to have a negative effect on the gumminess and chewiness [50]. Other studies in the literature reported a strong positive correlation between the fat content of cheeses and textural fracturability (r = 0.456, *p* < 0.05), hardness (r = 0.441, *p* < 0.05), adhesiveness (r = 0.684, *p* < 0.01) and gumminess (r = 0.721, *p* < 0.01) [51]. In addition, we observed a negative effect of fat content on the cohesion at 75% compression (r = 0.955, *p* < 0.01) (Table S1).

In this study, moisture exhibited a strong positive correlation with adhesiveness at 50% and 75% compression (r = 0.980 and r = 0.977, respectively). Additionally, at 50% compression, moisture exhibited a negative correlation with hardness (Table S1). The literature describes a positive relationship between cohesiveness and cheese moisture, such that the higher the water content, the greater the force exerted by the internal bonds of the cheese [50]. In this sense, CGC had a higher moisture content and therefore greater cohesiveness. However, MC, which had the lowest moisture content, had higher values of hardness and lower values of springiness than the other cheeses. Furthermore, a_w_ was positively correlated with hardness (r = 0.987) and adhesiveness (r = 0.989). On the other hand, hardness at 50% showed a negative correlation with adhesiveness (r = −0.999, *p* ≤ 0.01) and cohesiveness (r = −0.915). CGC had the highest bond strength and the lowest hardness. Cohesiveness showed a positive correlation with adhesiveness (r = 0.930, *p* > 0.05) and springiness (r = 0.980, *p* < 0.05) (Table S1). In general, in our study, springiness, cohesiveness or resilience were the parameters that showed the least variation from one cheese to another. However, other parameters such as hardness or gumminess showed more variability.

### 3.4. Microbiological Analysis

Table 6 presents the data from the microbiological counts carried out on goat cheeses from autochthonous goat breeds. In the goat cheeses studied, lactobacilli and lactococci were the most abundant microbial groups, as has been previously described in the literature. After the production and ripening processes of cheeses made from raw goat milk, the genus *Lactobacillus* was the most abundant microbial group [52]. MGC had the lowest counts of lactobacilli (6.53 log CFU/g of cheese) (*p* < 0.05). For the growth of lactococci, CGC and FC (8.03 and 8.00 log CFU/g of cheese, respectively) showed higher counts than MC (7.41 log CFU g of cheese) and MGC (5.99 log CFU/g of cheese), with significant differences (*p* > 0.05). In this study, a significant correlation was detected between lactobacilli counts and pH (r = 0.980). Lactobacilli may contribute to the acidification of milk during the initial steps of cheese making via the production of organic acids, mainly lactic acid [53].

There were no significant differences (*p* > 0.05) in the content of HLB between MC, MGC and FC, which all showed higher counts than CGC (6.58 log CFU/g cheese). Lactic acid bacteria (LAB) play an important role in inhibiting the growth of most undesirable microorganisms by acidifying the environment and contributing to the organoleptic characteristics of the cheese through processes such as lactose fermentation, protein hydrolysis and catabolic reactions during the ripening process [54]. Dairy products are important sources of biological active compounds of particular relevance to human health and of lactic acid bacteria. In addition, their influence on the development of the sensory characteristics of the cheese may be significant as they are one of the predominant microbial groups during the ripening process [7].

MGC had lower counts of total mesophilic aerobes (6.33 log CFU/g of cheese) than all other cheeses (*p* > 0.05). Finally, molds and yeasts were only detected in CGC, having been added during the elaboration process. For other raw goat milk cheeses, it has been suggested that the total FAA in cheeses might also be related to the presence of yeasts and their lipase activity [44]; on the contrary, in our study, we described a negative correlation between MUFAs (r = −0.960, *p* < 0.05) and PUFAs (r = −0.859) and molds and yeasts, although in this study, only was detected in CGC (Table S2). *Enterobacteriaceae* were only detected in MC. This type of bacteria is considered to be an indicator of the hygienic conditions of milk production [55] and high levels of *Enterobacteriace* in raw milk cheeses are of great concern for the dairy industry because of their technological and public health significance [56]. Artisanal cheeses are often manufactured from raw milk in farms or small dairies. Furthermore, the indigenous microbiota of raw milk is usually quite diverse and heterogeneous and has a significant impact on the overall microbiota of cheese with the starter used in the production process. Both factors were responsible for the differences between the cheeses [54].

The results obtained in this work showed that the microbiota of CGC was more abundant and diverse, and that MGC presented a lower total count than the other cheeses studied.

### 3.5. Sensory Analysis

The sensory properties of cheese are influenced by many factors, including species, milk production environment and processing technologies, as well as the chemical and microbiological properties of the raw materials used, which are among the main determinants of cheese yield and quality [57]. In addition to chemical composition and nutrient content, sensory evaluation is an important part of cheese quality assessment.

#### 3.5.1. Quantitative Descriptive Analysis by Trained Assessors

Table 7 shows the results of the sensory attributes (appearance, texture and olfactory–gustatory profile) of the cheeses studied in this work, evaluated by a panel of trained judges.

After evaluating the tactile and oral phases, no significant differences (*p* > 0.05) between the four cheeses analyzed in terms of springiness, surface roughness, friability, adherence and number of chews were detected. These results indicate that these attributes were not sufficient to differentiate the cheeses. The highest value for springiness was CGC (6.14), for surface roughness was MC (4.53), for friability was MGC (5.83), for adherence was FC (4.20) and for number of chews was MGC (12.86). With regard to springiness, CGC was described as the most elastic of all the cheeses studied. However, significant differences (*p* < 0.05) were detected by the panel in terms of the surface humidity between MC and the rest of the cheeses, in which a lower value was obtained (3.79). Moreover, a negative correlation between sensorial moisture and instrumental hardness was detected (r = −0.891) (Table S7). The cheese made with Cabra del Guadarrama milk had the highest surface humidity value (5.88), although there were no differences with MGC and FC (*p* > 0.05). This observation is in line with the results of the physico-chemical analysis previously carried out on the moisture content of each cheese. Statistically significant differences (*p* < 0.05) were found between the cheeses for the scores given by the assessors for firmness. In addition, both in the evaluation performed by texture analysis and by instrumental techniques, it was observed that MC had a higher firmness (6.44), which was related to its hardness (r = −0.987, *p* < 0.05). In addition, the firmness had an indirect relation with instrumental and sensory moisture (r = −1.00, *p* < 0.01 and r = −0.890, *p* < 0.05, respectively) and, on the other hand, with a_w_ (r = −0.986, *p* < 0.05) (Table S7). These results are consistent because juiciness depends directly on the water content of the food [29]. In relation to juiciness, significant differences (*p* < 0.05) were found between MC and MGC, which were perceived as having less juiciness than the other cheeses (2.36 and 2.76, respectively). A negative correlation between firmness and juiciness was detected (r = −0.982, *p* ≤ 0.05). In addition, a negative correlation between springiness and friability was detected (r = −0.923, *p* ≤ 0.05) (Tables S5–S7).

Regarding flavor attributes, no significant differences (*p* > 0.05) were found for salty, bitter and sweet flavors. However, significant differences were observed in the acid flavor between MC (3.82) and MGC (6.07) (*p* < 0.05). In relation to the salty flavor, MGC was the saltiest for the trained judges (4.94), according to the results of the physico-chemical analysis, although the differences observed were not statistically significant. In addition, a negative correlation between bitter flavor and adherence was detected (r = −0.965, *p* ≤ 0.05) (Tables S5–S7).

No significant differences in overall persistence and overall impression were found (*p* > 0.05). For this latter parameter MC was the best rated (6.96), followed by FC (6.86), MGC (6.58) and CGC (6.47). All cheeses were scored with values higher than 6 on a scale of 10 points, so the judges had high overall liking values of all the cheeses studied. The trained judges perceived that all the cheeses had a similar sensory quality.

A principal component analysis was performed, including physico-chemical parameters, instrumental texture parameters and sensory properties as variables. Three principal components were detected, with the first one explaining 43.80% of the explained variance, the second one 32.80% and the third one 23.34%. No other principal component was detected because the three of them explained 100% of the variance.

**Figure 3:**
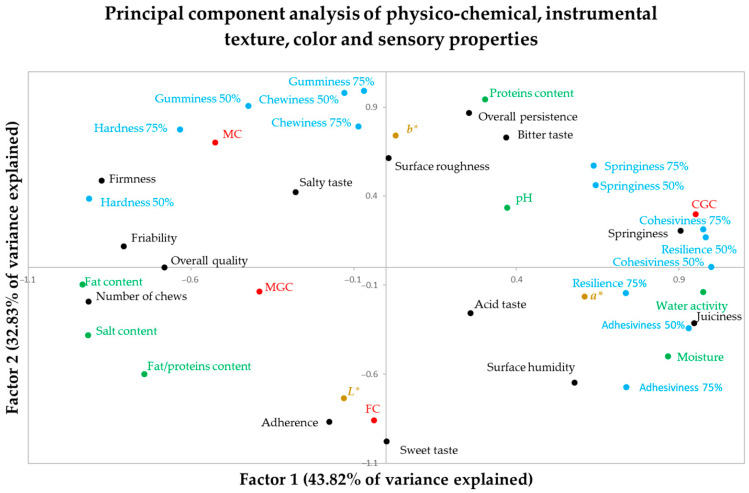
Distribution of the physico-chemical, instrumental texture, smell and sensory properties in the map configured by the first two principal components of goat cheeses made from the milk of different autochthonous goat breeds. CGC = Cabra del Guadarrama Cheese; MC = Malagueña Cheese; MGC = Murciano-Granadina Cheese; FC = Florida Cheese. Physico-chemical variates are written in green ink, instrumental texture variates are written in blue ink, color variates are written in brown ink, sensory variates are written in black ink and cheeses are written in red ink.

Table 8 shows the component matrix in which the three main components are detected and the correlation coefficients of each variable with each of the selected main components appear. The variables with the highest correlation with each of the principal components are shaded. Figure 3 shows the distribution of the physico-chemical, instrumental texture, color and sensory properties in the map configured by the first two principal components, which together explain the 73.39% of the observed variance.

The first component represents the water content (moisture, water activity) and related sensory variables (juiciness, springiness) or instrumental variables (springiness, adhesiveness). Similarly, hardness (both instrumental and sensory), fat content and other variables are negatively correlated with this main component (Table 8; Figure 3). This first principal component is what allows us to differentiate CGC from MGC and MC.

The second main component refers to the protein content and related properties. include instrumental texture (hardness, gumminess, chewiness) and sensory properties (such as persistence and bitterness, which are related to the cheeses included in this work with a higher protein content). On the contrary, it is negatively correlated with the sweet flavor and sensory adherence. This second factor is what mainly allows us to differentiate FC from CGC and MC (Table 8, Figure 3).

The third main component is the acidic character of the cheese. The acidic and salty flavors are positively correlated with this main component, and the pH is negatively correlated. This main component allows us to differentiate MGC, which is a more acidic cheese, from FC and MC.

**Figure 4:**
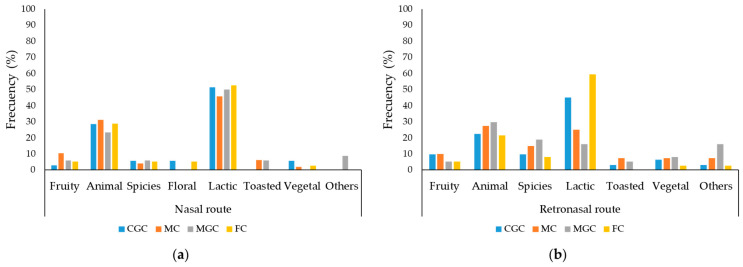
Aroma analysis characteristics evaluated by a trained panel by means of two methods: (**a**) nasal route and (**b**) retronasal route. The results are expressed as the percentage of panelists who detected smells from each olfactory family both nasally and retronasally. No significant differences were found (*p* > 0.05). CGC = Cabra del Guadarrama Cheese; MC = Malagueña Cheese; MGC = Murciano-Granadina Cheese; FC = Florida Cheese. No significant differences were found.

The olfactory profile of the cheeses was described by a panel of trained judges via two routes: the nasal route (Figure 4a) and the retronasal route (Figure 4b). The panelists detected the lactic and animal families as the dominant smells through the nasal route, followed by the fruity and spicy families. Of the lactic family, FC and CGC were the cheeses for which a higher percentage of judges detected their presence (52.63% and 51.43%, respectively). Animal smell was detected by 31.25% of the panelists in MC and by a similar percentage in CGC and FC (28.95% and 28.57%, respectively). Using the retronasal route, the panelists detected the smells more frequently, with the lactic and animal families being the ones that a higher percentage of judges identified. Fruit, spice and vegetable smells were detected more frequently in all cheeses by the retronasal route than by the nasal route. Via the retronasal route, the aromas of the lactic family were also perceived by a greater percentage of the panel judges (59.46%), followed by CGC (45.16%).

#### 3.5.2. Consumer Acceptance

**Figure 5:**
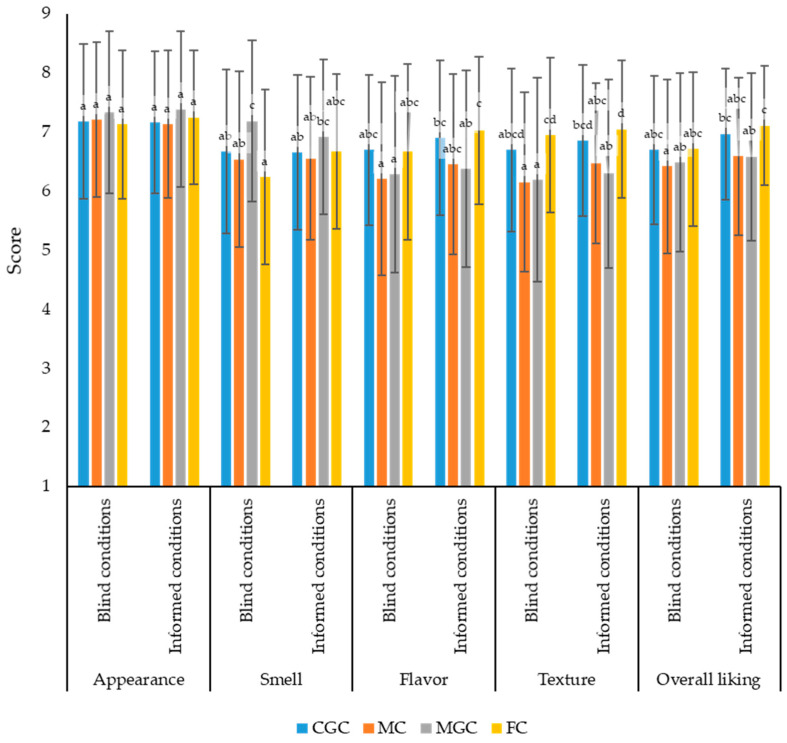
Hedonic test to rate the appearance, smell, flavor, texture and overall liking of goat cheeses made from the milk of different goat cheeses by consumers in both blind and informed conditions (n = 116). Different letters denote statistically significant differences (*p* ≤ 0.05).

Results from the hedonic analyses measured both in blind (uninformed) and informed conditions with untrained consumers (n = 116) are shown in Figure 5. The results of the acceptance tests with consumer judges showed that goat cheeses were moderately valued in the hedonic test (appearance, smell, flavor, texture and overall liking), with values above 6 points on a scale of 9 points. Several authors indicated a high overall liking in hedonic analyses when they obtained scores over 6 for different food products [58,59,60,61].

With respect to appearance, a higher score was obtained for MGC under blind and informed conditions (7.33 and 7.38, respectively) than rest of the cheeses, although no significant differences were found (*p* > 0.05). When consumers knew the characteristics of the cheeses (goat cheeses, raw milk cheeses and milk from native breeds from different regions of Spain), their assessment of the appearance of the cheeses did not change significantly.

In blind conditions, FC obtained lower smell scores than MGC (*p* < 0.05). However, under informed conditions, no significant differences were detected between the four cheeses studied. The information improved consumers’ assessment of the smell of FC.

In blind conditions, the judges did not give different scores for the flavor of the cheeses. In informed conditions, FC obtained better scores than MGC (*p* < 0.05). In the same way as for the evaluation of the smell, the information improved the evaluation of FC by consumers.

In both blind and informed conditions, the consumers gave better scores for texture acceptance for FC than MC and MGC (*p* < 0.05). Texture acceptance scores for CGC were also higher than for MC and MGC. In this case, however, the differences were not significant. As previously discussed, the textural properties of FC and CGC were more similar to each other than to those of MC and MGC, with the latter two being harder. The information did not significantly modify consumers’ assessment of the texture.

Finally, in blind conditions, there were no differences in overall liking between the cheeses studied. However, when consumers were informed, they preferred FC to MGC. Providing information to consumers worsened the overall liking of MGC.

A positive correlation was detected between appearance and smell (r = 0.954, *p* < 0.05). A positive correlation was detected between overall quality and moisture and adhesivity (r = 0.973 and r = 0.985, respectively, *p* < 0.05), and a negative correlation with hardness (r = −0.961, *p* < 0.05) (Table S8).

The results of the rank test, which was carried out in both blind and informed conditions, showed that there were no significant differences between the four cheeses studied. In the blind test, the FC was rated first, followed in order by MGC, CGC and MC. Under informed conditions, however, a change in the order of preference was observed among some of the cheeses: FC remained in first place and was still rated as the best by consumers. Second place went to the CGC, possibly because it was from a local breed from Madrid and was favored by the group of consumers who carried out the tasting. The MC and MGC obtained the lowest preference, meaning they were the cheeses most affected when the tasters knew the goat breed they came from. In any case, the differences were small, with no significant differences in the order of preference for the cheeses in either condition.

Furthermore, the lipolysis of triacylglycerols (TAGs) plays an essential role in the sensory properties of cheeses. Some FFAs have been shown to either directly contribute to the aroma characteristics of numerous cheeses or indirectly contribute as precursors of the compounds that are responsible for the aroma [62]. In this study, MUFA and UFA content showed a significant negative correlation (*p* < 0.05) with flavor (r = −0.967 and r = −0.992, respectively) (Table S5). Few previous studies have been able to correlate the sensory attributes of goat cheeses, smell and flavor, with FFAs [29].

The sensory properties of goat cheeses are an important factor for consumer acceptability and the marketability of the products. When cheese quality is analyzed on the market through surveys of consumer perceptions, selection, freshness and flavor were listed as the primary reasons for purchasing [63]. The results of the sensory analysis showed the impact of information on the origin of the product producing a positive effect on the results related to flavor, appearance, texture, smell and the quality of the product.

This study presents the characterization of four goat cheeses made in artisanal dairies with milk from different indigenous goat breeds. To the best of our knowledge, this is the first work that has studied the physico-chemical, microbiological and sensory characteristics of cheeses made from the autochthonous goat breed of Madrid, Cabra del Guadarrama, and compared them with those of other Spanish goat breeds. The results show the high quality of the cheeses, which contributes to the conservation of these breeds, and confirms the need to raise consumer awareness of indigenous breeds in order to increase the consumption of these products.

## 4. Conclusions

This is the first study to examine the physico-chemical, microbiological and sensory characteristics of a cheese made with milk from the Guadarrama goat breed, which is native to the Madrid region. This study also evaluates purchase intention and consumer acceptance, and compares the cheese’s properties with those of other Spanish goat breeds. Cabra del Guadarrama Cheese had a lower fat content than the rest of the cheeses studied. The fatty acid profile of Cabra del Guadarrama Cheese was more similar to Malagueña Cheese than to the Florida and Murciano-Granadina Cheeses. These two cheeses are produced in areas located just a few kilometers from each other. Cabra del Guadarrama Cheese was the one with the most different fatty acid profile compared to the rest of the cheeses studied.

The results demonstrate the high quality of the cheeses made from goat milk from the local breeds of Spain and the need to raise consumer awareness of autochthonous breed dairy products as a way to increase the consumption of these products, thereby helping to conserve them, which represents an important genetic heritage and contributes to the sustainability of Spain’s agricultural ecosystems.

## Figures and Tables

**Table 1:** Characteristics of the type of livestock and the cheeses from different autochthonous goat breeds.

	CGC	MC	MGC	FC
Location	Ávila	Madrid	Cádiz	Sevilla
Origin of the autochthonous breed	Madrid	Andalucía	Andalucía	Andalucía
Number of goats	105	170	150	NDa
Livestock feed	Extensive	Extensive	Extensive	Extensive
Origin of the milk	Own livestock	Own livestock	Own livestock	Different livestock
Starter culture	B and Y	B	B and Y	B
Type of brine	Immersed	Immersed	Dry	Immersed
Weight of the cheeses (kg)	1	1	2	0.8

CGC = Cabra del Guadarrama Cheese; MC = Malagueña Cheese; MGC = Murciano-Granadina Cheese; FC = Florida Cheese. B = bacteria, Y = yeast. NDa = no data available.

**Table 2:** Comparison of physico-chemical composition (fat, fat in dry matter (%), proteins, proteins in dry matter (%), fat/proteins ratio, salt (%), salt in dry matter (%), pH, moisture (%) and a_w_) of goat cheeses made from the milk of different autochthonous goat breeds.

	CGC	MC	MGC	FC
Physico-Chemical Composition				
Fat (%)	31.28 ± 0.62 ^a^	37.05 ± 2.09 ^c^	39.26 ± 0.70 ^d^	35.46 ± 1.18 ^b^
Fat in dry matter (%)	50.02 ± 0.99 ^a^	59.25 ± 3.34 ^c^	62.79 ± 1.12 ^d^	56.72 ± 1.88 ^b^
Proteins (%)	26.20 ± 0.35 ^c^	26.23 ± 0.42 ^c^	23.45 ± 0.16 ^b^	22.34 ± 0.67 ^a^
Proteins in dry matter (%)	41.90 ± 0.55 ^c^	41.96 ± 0.68 ^c^	37.51 ± 0.26 ^b^	35.74 ± 1.08 ^a^
Fat/proteins ratio	1.19 ± 0.03 ^a^	1.41 ± 0.06 ^b^	1.68 ± 0.02 ^d^	1.59 ± 0.03 ^c^
Salt (%)	1.25 ± 0.06 ^a^	1.72 ± 0.35 ^b^	1.87 ± 0.25 ^b^	1.78 ± 0.24 ^b^
Salt in dry matter (%)	2.00 ± 0.10 ^a^	2.99 ± 0.40 ^b^	2.75 ± 0.55 ^b^	2.62 ± 0.90 ^b^
pH	5.36 ± 0.05 ^c^	5.39 ± 0.04 ^c^	4.85 ± 0.05 ^a^	5.27 ± 0.08 ^b^
Moisture (%)	37.48 ± 3.02 ^b^	30.77 ± 2.03 ^a^	33.01 ± 0.69 ^a^	36.30 ± 1.81 ^b^
a_w_	0.9481 ± 0.003 ^d^	0.927 ± 0.004 ^a^	0.933 ± 0.002 ^b^	0.941 ± 0.004 ^c^

Results are reported as mean ± SD of 12 values from triplicate measurements of two different cheese samples elaborated in two independent trials. Different letters within the same row denote statistically significant differences (*p* ≤ 0.05). CGC = Cabra del Guadarrama Cheese; MC = Malagueña Cheese; MGC = Murciano-Granadina Cheese; FC = Florida Cheese; a_w_ = water activity.

**Table 3:** Fatty acid profile (mg FAME/g cheese) and health lipid indices of goat cheeses made from the milk of different autochthonous goat breeds measured by HPLC-MS.

Fatty Acids	CGC	MC	MGC	FC
Butyric acid (C4:0)	2.22 ± 0.12 ^a^	2.60 ± 0.12 ^bc^	2.83 ± 0.11 ^c^	2.46 ± 0.09 ^b^
Caproic acid (C6:0)	3.92 ± 0.19 ^a^	4.27 ± 0.35 ^b^	5.13 ± 0.13 ^c^	4.13 ± 0.10 ^ab^
Caprylic acid (C8:0)	5.98 ± 0.25 ^a^	6.24 ± 0.69 ^a^	7.95 ± 0.07 ^b^	6.14 ± 0.06 ^a^
Capric acid (C10:0)	22.86 ± 1.03 ^a^	22.79 ± 3.36 ^a^	29.96 ± 0.20 ^b^	23.01 ± 0.37 ^a^
Hendecanoic acid (C11:0)	0.28 ± 0.03 ^ab^	0.28 ± 0.08 ^b^	0.22 ± 0.01 ^ab^	0.20 ± 0.02 ^a^
Lauric acid (C12:0)	10.67 ± 0.68 ^a^	10.28 ± 1.67 ^a^	13.84 ± 0.08 ^b^	10.96 ± 0.36 ^a^
Myristic acid (C14:0)	22.12 ± 0.82 ^a^	25.09 ± 2.57 ^bc^	27.79 ± 0.10 ^c^	24.14 ± 0.31 ^ab^
Myristoleic acid (C14:1n5)	0.25 ± 0.01 ^a^	0.24 ± 0.01 ^a^	0.30 ± 0.00 ^b^	0.31 ± 0.01 ^b^
Pentadecylic acid (C15:0)	2.03 ± 0.06 ^a^	2.37 ± 0.26 ^b^	1.76 ± 0.02 ^a^	1.82 ± 0.07 ^a^
Palmitic acid (C16:0)	60.47 ± 1.77 ^a^	67.72 ± 4.56 ^b^	85.58 ± 1.66 ^c^	72.67 ± 1.06 ^b^
Palmitoleic acid (C16:1n7)	1.09 ± 0.01 ^a^	1.53 ± 0.06 ^b^	1.63 ± 0.07 ^b^	1.85 ± 0.05 ^c^
Margaric acid (C17:0)	1.58 ± 0.02 ^b^	2.46 ± 0.07 ^c^	1.34 ± 0.00 ^a^	1.45 ± 0.02 ^b^
Stearic acid (C18:0)	28.97 ± 0.27 ^a^	36.13 ± 1.25 ^b^	35.68 ± 0.61 ^b^	33.06 ± 2.30 ^b^
Cis-Vaccenic acid (C18:1n7c)	0.60 ± 0.02 ^a^	0.99 ± 0.06 ^b^	0.93 ± 0.03 ^b^	0.99 ± 0.06 ^b^
Oleic acid (C18:1n9c)	47.90 ± 0.57 ^a^	55.94 ± 2.97 ^b^	55.07 ± 1.53 ^b^	58.06 ± 2.87 ^b^
Linoleic acid (C18:2n6c)	4.92 ± 0.08 ^a^	8.47 ± 0.15 ^c^	10.50 ± 0.51 ^d^	7.06 ± 0.37 ^b^
α-Linolenic acid (C18:3n3)	1.18 ± 0.04 ^b^	1.91 ± 0.11 ^c^	0.78 ± 0.03 ^a^	1.21 ± 0.08 ^b^
Arachidic acid (C20:0)	1.56 ± 0.04 ^c^	2.38 ± 0.32 ^d^	0.57 ± 0.01 ^a^	0.96 ± 0.06 ^b^
C20:1n9	0.11 ± 0.01 ^a^	0.22 ± 0.01 ^d^	0.21 ± 0.00 ^c^	0.17 ± 0.01 ^b^
Arachidonic acid (C20:4n6)	0.36 ± 0.00 ^a^	0.49 ± 0.02 ^b^	0.49 ± 0.01 ^b^	0.56 ± 0.03 ^c^
Timnodonic acid-EPA (C20:5n3)	n.d.	0.15 ± 0.01	n.d.	n.d.
C21:0	0.20 ± 0.00 ^a^	0.40 ± 0.05 ^b^	n.d.	n.d.
Behenic acid (C22:0)	0.48 ± 0.00 ^c^	0.73 ± 0.10 ^d^	0.18 ± 0.00 ^a^	0.28 ± 0.01 ^b^
Clupanodonic acid-DPA (C22:5n3)	0.23 ± 0.01 ^b^	0.36 ± 0.01 ^d^	0.16 ± 0.00 ^a^	0.27 ± 0.01 ^c^
C23:0	n.d.	0.15 ± 0.02	n.d.	n.d.
Lignoceric acid (C24:0)	0.14 ± 0.01 ^a^	0.23 ± 0.02 ^b^	n.d.	n.d.
TOTAL	220.12 ± 4.72 ^a^	254.42 ± 12.81 ^b^	282.90 ± 4.77 ^c^	251.78 ± 5.83 ^b^
Health Lipid Indices				
ΣSCFA	34.87 ± 1.30 ^a^	37.36 ± 4.70 ^a^	45.83 ± 0.41 ^b^	36.57 ± 1.68 ^a^
ΣSUFA	162.73 ± 4.26 ^a^	189.22 ± 16.15 ^b^	212.03 ± 2.69 ^c^	184.47 ± 6.74 ^b^
ΣMUFA	49.36 ± 1.28 ^a^	58.19 ± 2.92 ^b^	57.64 ± 1.65 ^b^	62.56 ± 3.37 ^b^
ΣPUFA	6.64 ± 0.14 ^a^	11.42 ± 0.18 ^c^	11.77 ± 0.54 ^c^	9.24 ± 0.37 ^b^
UFAs	56.00 ± 1.38 ^a^	69.60 ± 2.86 ^b^	69.41 ± 2.19 ^b^	71.80 ± 3.74 ^b^
∑n6/∑n-3	3.73 ± 0.05 ^a^	3.66 ± 0.20 ^a^	11.56 ± 0.30 ^c^	4.85 ± 0.74 ^b^
AI	2.84 ± 0.11 ^ab^	2.65 ± 0.35 ^ab^	3.03 ± 0.07 ^c^	2.55 ± 0.11 ^a^
TI	3.59 ± 0.08 ^a^	3.35 ± 0.31 ^a^	4.08 ± 0.06 ^b^	3.38 ± 0.07 ^a^
DFA	84.68 ± 2.01 ^a^	106.13 ± 2.12 ^b^	104.85 ± 2.86 ^b^	105.51 ± 6.00 ^b^
OFA	92.97 ± 2.71 ^a^	106.04 ± 9.29 ^b^	126.69 ± 1.65 ^c^	109.24 ± 3.04 ^b^
H/H	0.57 ± 0.02 ^ab^	0.62 ± 0.08 ^b^	0.52 ± 0.01 ^a^	0.62 ± 0.02 ^b^

Results are reported as the mean ± SD of 12 values from triplicate measurements of two different cheese samples elaborated in two independent trials. Different letters within the same row denote statistically significant differences (*p* ≤ 0.05). FAME = fatty acid methyl esters; CGC = Cabra del Guadarrama Cheese; MC = Malagueña Cheese; MGC = Murciano-Granadina Cheese; FC = Florida Cheese; n.d. = none detected; Health Lipid Indices = ΣSCFA = all short-chain fatty acids (C4:0, C6:0, C8:0, C10:0); ΣSUFA = all saturated fatty acids; ΣMUFA = all monounsaturated fatty acids; PUFAs = all polyunsaturated fatty acids; UFAs = all unsaturated fatty acids (ΣMUFA + ΣPUFA); ∑n6/∑n-3 ratio; AI = index of atherogenicity; TI = index of thrombogenicity; DFA = hypocholesterolemic fatty acids (ΣUFA + C18:0); OFA = hypercholesterolemic fatty acids (Σ SUFA-C18:0); H/H = hypocholesterolemic/hypercholesterolemic ratio.

**Table 4:** Fatty acid profile (% of total fatty acids) of goat cheeses made from the milk of different autochthonous goat breeds measured by HPLC-MS.

Fatty Acids (%)	CGC	MC	MGC	FC
Butyric acid (C4:0)	1.01 ± 0.03 ^a^	1.01 ± 0.02 ^a^	1.00 ± 0.02 ^a^	0.98 ± 0.02 ^a^
Caproic acid (C6:0)	1.78 ± 0.04 ^b^	1.69 ± 0.05 ^a^	1.82 ± 0.02 ^b^	1.65 ± 0.02 ^a^
Caprylic acid (C8:0)	2.72 ± 0.05 ^b^	2.49 ± 0.15 ^a^	2.82 ± 0.04 ^b^	2.45 ± 0.04 ^a^
Capric acid (C10:0)	10.43 ± 0.25 ^b^	9.20 ± 0.88 ^a^	10.63 ± 0.13 ^b^	9.19 ± 0.29 ^a^
Hendecanoic acid (C11:0)	0.13 ± 0.01 ^b^	0.12 ± 0.03 ^b^	0.08 ± 0.00 ^a^	0.08 ± 0.01 ^a^
Lauric acid (C12:0)	4.88 ± 0.19 ^bc^	4.16 ± 0.46 ^a^	4.92 ± 0.11 ^c^	4.35 ± 0.19 ^ab^
Myristic acid (C14:0)	10.09 ± 0.16 ^a^	10.01 ± 0.53 ^a^	9.88 ± 0.19 ^a^	9.62 ± 0.26 ^a^
Myristoleic acid (C14:1n5)	0.11 ± 0.00 ^b^	0.10 ± 0.00 ^a^	0.11 ± 0.00 ^b^	0.13 ± 0.01 ^c^
Pentadecylic acid (C15:0)	0.93 ± 0.01 ^c^	0.94 ± 0.06 ^c^	0.63 ± 0.02 ^a^	0.73 ± 0.04 ^b^
Palmitic acid (C16:0)	27.53 ± 0.22 ^b^	26.74 ± 0.47 ^a^	30.21 ± 0.11 ^d^	28.67 ± 0.47 ^c^
Palmitoleic acid (C16:1n7)	0.51 ± 0.02 ^a^	0.59 ± 0.05 ^b^	0.57 ± 0.02 ^ab^	0.72 ± 0.04 ^c^
Margaric acid (C17:0)	0.72 ± 0.01 ^c^	0.96 ± 0.02 ^d^	0.47 ± 0.01 ^a^	0.58 ± 0.02 ^b^
Stearic acid (C18:0)	13.11 ± 0.27 ^a^	14.12 ± 0.28 ^b^	12.6 ± 0.03 ^a^	13.14 ± 0.52 ^a^
Cis-Vaccenic acid (C18:1n7c)	0.27 ± 0.01 ^a^	0.38 ± 0.04 ^b^	0.33 ± 0.01 ^b^	0.38 ± 0.02 ^b^
Oleic acid (C18:1n9c)	21.63 ± 0.59 ^ab^	21.42 ± 2.15 ^ab^	19.39 ± 0.23 ^a^	23.11 ± 0.54 ^b^
Linoleic acid (C18:2n6c)	2.23 ± 0.02 ^a^	3.28 ± 0.19 ^c^	3.67 ± 0.12 ^d^	2.76 ± 0.11 ^b^
α-Linolenic acid (C18:3n3)	0.54 ± 0.01 ^b^	0.75 ± 0.01 ^c^	0.28 ± 0.00 ^a^	0.51 ± 0.07 ^b^
Arachidic acid (C20:0)	0.70 ± 0.03 ^c^	0.96 ± 0.08 ^d^	0.20 ± 0.00 ^a^	0.41 ± 0.07 ^b^
C20:1n9	0.05 ± 0.00 ^a^	0.09 ± 0.00 ^d^	0.07 ± 0.00 ^c^	0.07 ± 0.00 ^b^
Arachidonic acid (C20:4n6)	0.16 ± 0.00 ^a^	0.19 ± 0.00 ^b^	0.17 ± 0.00 ^a^	0.22 ± 0.01 ^c^
Timnodonic acid-EPA (C20:5n3)	n.d.	0.06 ± 0.00 ^b^	n.d.	n.d.
C21:0	0.09 ± 0.00 ^b^	0.16 ± 0.01 ^c^	n.d.	n.d.
Behenic acid (C22:0)	0.22 ± 0.01 ^c^	0.29 ± 0.02 ^d^	0.06 ± 0.00 ^a^	0.12 ± 0.03 ^b^
Clupanodonic acid-DPA (C22:5n3)	0.10 ± 0.01 ^b^	0.14 ± 0.00 ^c^	0.06 ± 0.00 ^a^	0.11 ± 0.01 ^b^
C23:0	n.d.	n.d.	n.d.	n.d.
Lignoceric acid (C24:0)	0.06 ± 0.00 ^b^	0.09 ± 0.01 ^c^	n.d.	n.d.
TOTAL	100.00	100.00	100.00	100.00

Results are reported as the mean ± SD of 12 values from triplicate measurements of two different cheese samples elaborated in two independent trials. Different letters within the same row denote statistically significant differences (*p* ≤ 0.05). HPLC-MS = high-performance liquid chromatography coupled to mass spectrometry; CGC = Cabra del Guadarrama Cheese; MC = Malagueña Cheese; MGC = Murciano-Granadina Cheese; FC = Florida Cheese; n.d.: none detected.

**Table 5:** Comparison of the color and texture of goat cheeses made from the milk of different autochthonous goat breeds.

		CGC	MC	MGC	FC
Color					
Paste					
	L*	81.34 ± 2.39 ^a^	77.34 ± 8.06 ^a^	89.98 ± 4.96 ^b^	86.16 ± 2.28 ^b^
	a*	−2.09 ± 1.54 ^a^	−2.88 ± 1.43 ^a^	−2.15 ± 1.90 ^a^	−2.58 ± 0.33 ^a^
	b*	15.17 ± 0.76 ^c^	18.16 ± 1.82 ^d^	10.87 ± 2.05 ^a^	12.89 ± 0.89 ^b^
Cheese rind					
	L*	55.80 ± 9.55 ^a^	66.22 ± 7.85 ^bc^	63.57 ± 4.49 ^b^	70.99 ± 2.36 ^c^
	a*	2.85 ± 2.49 ^b^	2.97 ± 1.45 ^b^	9.08 ± 2.34 ^c^	−2.48 ± 0.65 ^a^
	b*	16.62 ± 4.82 ^a^	19.95 ± 3.88 ^b^	24.64 ± 2.46 ^c^	15.98 ± 1.22 ^a^
Texture					
Hardness (N)	50%	46.07 ± 6.16 ^a^	76.06 ± 14.90 ^b^	70.86 ± 10.99 ^b^	53.91 ± 7.52 ^a^
	75%	37.39 ± 6.81 ^a^	58.09 ± 13.01 ^c^	45.10 ± 5.45 ^b^	33.49 ± 6.17 ^a^
Springiness	50%	0.87 ± 0.03 ^b^	0.79 ± 0.10 ^ab^	0.83 ± 0.13 ^ab^	0.76 ± 0.13 ^a^
	75%	0.83 ± 0.09 ^c^	0.71 ± 0.16 ^ab^	0.75 ± 0.11 ^bc^	0.64 ± 0.10 ^a^
Cohesiveness	50%	0.53 ± 0.03 ^b^	0.38 ± 0.06 ^a^	0.39 ± 0.06 ^a^	0.43 ± 0.06 ^a^
	75%	0.35 ± 0.06 ^b^	0.26 ± 0.06 ^a^	0.25 ± 0.04 ^a^	0.27 ± 0.05 ^a^
Adhesiveness (Ns)	50%	0.28 ± 0.21 ^b^	0.02 ± 0.03 ^a^	0.06 ± 0.07 ^a^	0.20 ± 0.15 ^b^
	75%	0.17 ± 0.17 ^b^	0.01 ± 0.04 ^a^	0.08 ± 0.15 ^ab^	0.18 ± 0.21 ^b^
Chewiness (N)	50%	21.19 ± 2.56 ^ab^	23.30 ± 7.75 ^b^	20.68 ± 6.55 ^ab^	16.45 ± 5.57 ^a^
	75%	10.77 ± 2.40 ^b^	11.18 ± 5.83 ^b^	11.96 ± 3.40 ^b^	5.59 ± 2.26 ^a^
Resilience	50%	0.33 ± 0.03 ^b^	0.20 ± 0.03 ^a^	0.21 ± 0.05 ^a^	0.22 ± 0.04 ^a^
	75%	0.20 ± 0.05 ^b^	0.12 ± 0.03 ^a^	0.18 ± 0.04 ^b^	0.15 ± 0.03 ^a^
Gumminess (N)	50%	24.48 ± 3.08 ^ab^	29.56 ± 9.19 ^b^	25.16 ± 9.11 ^ab^	21.46 ± 7.97 ^a^
	75%	12.99 ± 2.81 ^bc^	15.27 ± 6.03 ^c^	11.24 ± 2.54 ^ab^	8.88 ± 2.05 ^a^

Results are reported as the mean ± SD of 20 values from five replicate measurements of two different cheese samples elaborated in two independent trials. Different letters within the same row denote statistically significant differences (*p* ≤ 0.05). CGC = Cabra del Guadarrama Cheese; MC = Malagueña Cheese; MGC = Murciano-Granadina Cheese; FC = Florida Cheese. L* = lightness; a* = red index; b* = yellow index.

**Table 6:** Comparison of the microorganism counts (log CFU/g) of goat cheeses made from the milk of different autochthonous goat breeds.

Microorganism (log CFU/g)	CGC	MC	MGC	FC
Lactobacilli	9.10 ± 0.06 ^b^	9.09 ± 0.03 ^b^	6.53 ± 0.21 ^a^	9.08 ± 0.07 ^b^
Lactococci	8.03 ± 0.09 ^c^	7.41 ± 0.02 ^b^	5.99 ± 0.11 ^a^	8.00 ± 0.16 ^c^
HLB	6.58 ± 0.69 ^a^	7.38 ± 0.17 ^b^	5.93 ± 0.06 ^a^	6.60 ± 0.04 ^a^
MAB	7.80 ± 0.13 ^b^	7.52 ± 0.48 ^b^	6.33 ± 0.10 ^a^	7.99 ± 0.10 ^b^
Molds	2.31 ± 0.21	n.d.	n.d.	n.d.
Yeast	2.51 ± 0.73	n.d.	n.d.	n.d.
Enterobacteriaceae	n.d.	1.70 ± 0.65	n.d.	n.d.

Results are reported as the mean ± SD of eight values from duplicate measurements of two different cheese samples elaborated in two independent trials. Different letters within the same row denote statistically significant differences (*p* ≤ 0.05). CGC = Cabra del Guadarrama Cheese; MC = Malagueña Cheese; MGC = Murciano-Granadina Cheese; FC = Florida Cheese; n.d.: none detected; CFU = Colony Forming Units; HLB = heterofermentative lactic bacteria; MAB = mesophilic aerobic bacteria.

**Table 7:** Sensory analysis (quantitative descriptive analysis) of goat cheeses made from the milk of different autochthonous goat breeds. The intensity of different parameters was evaluated by a panel of trained assessors (n = 14).

Phase	Sensory Attributes	Scale ^1^	CGC	MC	MGC	FC
Tactile phase	Springiness	Nil to high	6.14 ± 1.72 ^a^	4.66 ± 2.87 ^a^	3.84 ± 2.18 ^a^	4.85 ± 2.37 ^a^
	Surface roughness	Smooth to sandy	3.84 ± 1.85 ^a^	4.53 ± 2.01 ^a^	2.91 ± 1.71 ^a^	3.60 ± 2.29 ^a^
	Surface humidity	Dry to wet	5.88 ± 1.85 ^b^	3.79 ± 1.37 ^a^	5.75 ± 1.83 ^b^	5.71 ± 1.63 ^b^
Bucal phase	Firmness	Dry to wet	3.95 ± 1.97 ^a^	6.44 ± 1.22 ^c^	5.61 ± 1.84 ^bc^	4.44 ± 1.19 ^ab^
	Friability	Nil to high	4.46 ± 1.75 ^a^	5.28 ± 1.89 ^a^	5.83 ± 1.57 ^a^	4.74 ± 1.20 ^a^
	Adherence	Nil to high	3.37 ± 1.67 ^a^	3.47 ± 1.53 ^a^	3.50 ± 1.79 ^a^	4.20 ± 1.41 ^a^
	Juiciness	Dry to juicy	4.30 ± 2.05 ^b^	2.36 ± 0.98 ^a^	2.76 ± 1.64 ^ab^	3.61 ± 1.63 ^ab^
	Number of chews	Number	9.85 ± 3.05 ^a^	11.86 ± 5.14 ^a^	12.86 ± 7.64 ^a^	11.57 ± 4.73 ^a^
	Acid flavor	Lower to higher	5.30 ± 1.84 ^ab^	3.82 ± 2.11 ^a^	6.07 ± 1.65 ^b^	4.56 ± 2.13 ^ab^
	Salty flavor	Lower to higher	4.42 ± 2.01 ^a^	4.47 ± 2.10 ^a^	4.94 ± 1.68 ^a^	4.00 ± 1.50 ^a^
	Bitter flavor	Lower to higher	3.79 ± 2.85 ^a^	3.39 ± 2.75 ^a^	3.56 ± 2.15 ^a^	2.73 ± 1.82 ^a^
	Sweet flavor	Lower to higher	1.11 ± 1.09 ^a^	1.03 ± 0.85 ^a^	1.18 ± 1.52 ^a^	1.46 ± 1.33 ^a^
Olfactory–taste phase	Overall persistence	Lower to higher	6.49 ± 1.81 ^a^	6.28 ± 1.92 ^a^	6.21 ± 1.10 ^a^	5.35 ± 1.62 ^a^
	Overall impression	Lower to higher	6.47 ± 1.36 ^a^	6.96 ± 1.58 ^a^	6.58 ± 2.05 ^a^	6.86 ± 1.45 ^a^

Results are reported as the mean ± SD of 14 values from of 14 panelists who evaluated each sample. Different letters within an attribute denote statistically significant differences (*p* ≤ 0.05). CGC = Cabra del Guadarrama Cheese; MC = Malagueña Cheese; MGC = Murciano-Granadina Cheese; FC = Florida Cheese. ^1^: Foods as their reference scale: springiness—butter, stuffed olive and sausage; surface roughness—apple and different biscuit cuts; surface humidity—walnut shell, orange rind, inside of banana peel and cut apple; firmness—spreadable cheese, sausage and cooked carrot; friability—boiled egg white, muffin and wafer; adherence—boiled egg white, boiled egg yolk and toffee caramel; and juiciness—biscotte, banana, apple, orange and watermelon. For persistence, the lowest third of the scale (0–3.33 points) will be used if the sensation persists for 5 s or less; the middle third (3.34–6.66 points) if it persists for between 5 and 10 s; and the highest third (6.67–20 points) if it persists for more than 10 s.

**Table 8:** Principal component analysis of the physico-chemical, instrumental texture and sensory variables of goat cheeses.

Component Matrix			
	Factor 1	Factor 2	Factor 3
CGC	0.86	0.50	0.08
FC	0.15	−0.85	−0.51
MC	−0.67	0.57	−0.48
MGC	−0.35	−0.22	0.91
Fat	−0.89	−0.30	0.35
Proteins	0.09	0.99	−0.14
Fat/proteins	−0.59	−0.75	0.30
Salt	−0.81	−0.57	0.12
pH	0.29	0.41	−0.86
Moisture	0.95	−0.30	−0.03
Water activity	0.98	0.08	−0.18
Color L* paste	0.03	−0.75	0.66
Color a* paste	0.63	−0.03	0.77
Color b* paste	−0.13	0.73	−0.67
Hardness 50%	−0.97	0.17	0.14
Hardness 75%	−0.79	0.61	−0.05
Springiness 50%	0.53	0.59	0.61
Springiness 75%	0.50	0.70	0.52
Cohesiveness 50%	0.97	0.22	−0.03
Cohesiveness 75%	0.90	0.42	−0.07
Adhesiveness 50%	0.98	−0.13	−0.14
Adhesiveness 75%	0.87	−0.49	−0.06
Chewiness 50%	−0.34	0.93	0.16
Chewiness 75%	−0.26	0.75	0.61
Resilience 50%	0.92	0.38	0.08
Resilience75%	0.75	0.02	0.66
Gumminess 50%	−0.61	0.79	−0.05
Gumminess 75%	−0.28	0.95	−0.11
Springiness	0.84	0.40	−0.37
Surface roughness	−0.13	0.60	−0.79
Surface humidity	0.71	−0.51	0.49
Firmness	−0.96	0.28	0.02
Friability	−0.81	−0.06	0.58
Adherence	0.02	−0.88	−0.47
Juiciness	0.99	−0.10	−0.06
Number of chews	−0.85	−0.39	0.35
Acid flavor	0.31	−0.19	0.93
Salty flavor	−0.36	0.35	0.86
Bitter flavor	0.20	0.79	0.58
Sweet flavor	0.22	−0.95	−0.21
Overall persistence	0.06	0.90	0.43
Overall quality	−0.66	−0.15	−0.73

CGC = Cabra del Guadarrama Cheese; MC = Malagueña Cheese; MGC = Murciano-Granadina Cheese; FC = Florida Cheese.

## Data Availability

The original contributions presented in this study are included in the article. Further information can be available after inquiries to the corresponding author.

## Supplementary Materials

The following supporting information can be downloaded at: https://www.mdpi.com/article/10.3390/foods14132368/s1, Table S1: Correlations coefficients between physico-chemical, color and texture instrumental variables; Table S2: Correlations coefficients between nutritional, physico-chemical, color, microorganism counts, sum of fatty acids profile and health lipid indices variables; Table S3: Correlations coefficients between texture instruments, sum of fatty acids profile and health lipid indices variables; Table S4: Correlations coefficients between nutritional, physico-chemical, color and sensorial variables; Table S5: Correlations coefficients between sensorial, sum of fatty acids profile and health lipid indices variables; Table S6: Correlations coefficients between microorganism counts and sensorial variables; Table S7: Correlations coefficients between texture instrumental and sensorial panel variables; Table S8: Correlations coefficients between texture instrumental and sensorial consumers variables.

## Abbreviations

The following abbreviations are used in this manuscript:

AI	Index of atherogenicity
a_w_	Water activity
CFU	Colony Forming Units
CGC	Cabra del Guadarrama Cheese
DFA	Hypocholesterolemic fatty acids
FAMEs	Fatty acid methyl esters
FFAs	Fatty acids
FC	Florida Cheese
H/H	Hypocholesterolemic and hypercholesterolemic FA ratio
HLB	Heterofermentative lactic bacteria
HPLC-MS	High-performance liquid chromatography coupled to mass spectrometry
IMIDRA	Instituto Madrileño de Investigación y Desarrollo Rural Agrario y Alimentario
MAB	Mesophilic aerobic bacteria
MC	Malagueña Cheese
MGC	Murciano-Granadina Cheese
MRS	Man Rogosa Sharpe
MUFAs	All monounsaturated fatty acids
n.d.	None detected
NDa	No data available
PCA	Plate count milk agar
PCA	Principal component analysis
PDA	Potato agar dextrose
PUFAs	All polyunsaturated fatty acids
OFAs	Hypercholesterolemic fatty acids
QDA	Quantitative descriptive analysis
SCFAs	All short-chain fatty acids
SD	Standard deviation
SFs	Saturated fatty acids
SUFAs	All saturated fatty acids
TI	Index of thrombogenicity
TAGs	Triacylglycerols
TPA	Texture profile analysis
UFAs	All unsaturated fatty acids
VRBG	Violet red bile glucose agar

## References

[1] MAPA (Ministerio de Agricultura, Pesca y Alimentación) Informe Anual de la Industria Alimentaria Española Periodo 2022–2023. https://www.mapa.gob.es/es/alimentacion/temas/industria-agroalimentaria/20240126informeanualindustria2022-20234t23ok_tcm30-659567.pdf.

[2] FAO (Food and Agriculture Organization of the United Nations) Domestic Animal Diversity Information System (DAD-IS). http://www.fao.org/dad-is/browse-by-country-and-species/en/%0Ahttp://www.fao.org/dad-is/en/.

[3] Fresno M., Torres A., Capote J., Álvarez S. (2020). Effect of Breed on Physicochemical and Sensory Characteristics of Fresh, Semihard and Hard Goat’s Milk Cheeses. J. Appl. Anim. Res..

[4] Hayaloglu A.A., Tolu C., Yasar K. (2013). Influence of Goat Breeds and Starter Culture Systems on Gross Composition and Proteolysis in Gokceada Goat Cheese during Ripening. Small Rumin. Res..

[5] ALKaisy Q.H., Al-Saadi J.S., AL-Rikabi A.K.J., Altemimi A.B., Hesarinejad M.A., Abedelmaksoud T.G. (2023). Exploring the Health Benefits and Functional Properties of Goat Milk Proteins. Food Sci. Nutr..

[6] Ramírez-Navas J.S., Aguirre-Londoño J., Aristizabal-Ferreira V.A., Castro-Narváez S. (2016). La Sal En El Queso: Diversas Interacciones. Agron. Mesoam..

[7] Kováčová M., Výrostková J., Dudriková E., Zigo F., Semjon B., Regecová I. (2021). Assessment of Quality and Safety of Farm Level Produced Cheeses from Sheep and Goat Milk. Appl. Sci..

[8] Currò S., Manuelian C.L., De Marchi M., Goi A., Claps S., Esposito L., Neglia G. (2020). Italian Local Goat Breeds Have Better Milk Coagulation Properties than Cosmopolitan Breed. Ital. J. Anim. Sci..

[9] Cruz Maceín J.L., Iriondo DeHond M., Miguel E. (2019). Cheese Consumption Culture in Central Spain (Madrid Region): Drivers and Consumer Profile. Br. Food J..

[10] MAPA (Ministerio de Agricultura, Pesca y Alimentación Análisis de la Estructura Productiva del Sector Caprino de Leche en España. https://cpage.mpr.gob.es/.

[11] (2006). AOAC (Association of Analytical Communities) Official Methods of the Analysis of the AOAC International—960.39.

[12] (2000). AOAC (Association of Analytical Communities) Official Methods of the Analysis of AOAC International—992.15.

[13] (2005). AOAC (Association of Analytical Communities) Official Methods of the Analysis of the AOAC International—925.10.

[14] Lee M.R.F., Tweed J.K.S., Kim E.J., Scollan N.D. (2012). Beef, Chicken and Lamb Fatty Acid Analysis—A Simplified Direct Bimethylation Procedure Using Freeze-Dried Material. Meat Sci..

[15] Paszczyk B., Łuczyńska J. (2020). The Comparison of Fatty Acid Composition and Lipid Quality Indices in Hard Cow, Sheep, and Goat Cheeses. Foods.

[16] Macdougall D.B. (2010). Colour Measurement of Food: Principles and Practice. Colour Meas. Princ. Adv. Ind. Appl..

[17] Delgado F.J., González-Crespo J., Cava R., Ramírez R. (2011). Proteolysis, Texture and Colour of a Raw Goat Milk Cheese throughout the Maturation. Eur. Food Res. Technol..

[18] Miguel E., Álvarez-Teno A., Iriondo de Hond M., Mancho C. (2015). Caracterización Sensorial de los Quesos de Madrid. Diferencias en la Percepción Sensorial y Utilidad del Análisis Sensorial para la Descripción de las Propiedades de Textura de los Quesos. ITEA, Volumen Extra. XVI Jornadas Sobre Producción Animal.

[19] BOE (Boletín Oficial del Estado) (2006). Real Decreto 1113/2006 de 29 de Septiembre, por el que se Aprueban las Normas de Calidad para Quesos y Quesos Fundidos.

[20] de la Haba Ruiz M.A., Ruiz Pérez-Cacho P., Dios Palomares R., Galán-Soldevilla H. (2016). Classification of Artisanal Andalusian Cheeses on Physicochemical Parameters Applying Multivariate Statistical Techniques. Dairy Sci. Technol..

[21] López Ruiz Á.L., Ruiz Morales F.d.A., Ruiz Pérez-Cacho P., Galán-Soldevilla H. (2023). Effect of Lactose-Reduction in Murciano-Granadina Semi-Hard Goat Cheese on Physicochemical and Sensory Characteristics. Foods.

[22] Di Trana A., Di Rosa A.R., Addis M., Fiori M., Di Grigoli A., Morittu V.M., Spina A.A., Claps S., Chiofalo V., Licitra G. (2022). The Quality of Five Natural, Historical Italian Cheeses Produced in Different Months: Gross Composition, Fat-Soluble Vitamins, Fatty Acids, Total Phenols, Antioxidant Capacity, and Health Index. Animals.

[23] MAPA (Ministerio de Agricultura, Pesca y Alimentación) Sistema Nacional de Información de Razas (ARCA). https://www.mapa.gob.es/es/ganaderia/temas/zootecnia/razas-ganaderas/default.aspx.

[24] Danezis G.P., Tsiplakou E., Pappa E.C., Pappas A.C., Mavrommatis A., Sotirakoglou K., Georgiou C.A., Zervas G. (2020). Fatty Acid Profile and Physicochemical Properties of Greek Protected Designation of Origin Cheeses, Implications for Authentication. Eur. Food Res. Technol..

[25] Pinho O., Mendes E., Alves M.M., Ferreira I.M. (2004). Chemical, Physical, and Sensorial Characteristics of “Terrincho” Ewe Cheese: Changes During Ripening and Intravarietal Comparison. J. Dairy Sci..

[26] Ruvalcaba-Gómez J.M., Ruiz-Espinosa H., Arteaga-Garibay R.I., Rojas-López M., Amador-Espejo G.G., Anaya-Esparza L.M., Delgado-Macuil R.J. (2020). Texture, Physicochemical and Sensory Properties of Artisanal Adobera Cheese from Los Altos de Jalisco, a Genuine Mexican Cheese. Int. J. Dairy Technol..

[27] Trmčić A., Ralyea R., Meunier-Goddik L., Donnelly C., Glass K., D’Amico D., Meredith E., Kehler M., Tranchina N., McCue C. (2017). Consensus Categorization of Cheese Based on Water Activity and PH—A Rational Approach to Systemizing Cheese Diversity. J. Dairy Sci..

[28] Ioannidou M.D., Maggira M., Samouris G. (2022). Physicochemical Characteristics, Fatty Acids Profile and Lipid Oxidation during Ripening of Graviera Cheese Produced with Raw and Pasteurized Milk. Foods.

[29] Gámbaro A., González V., Jiménez S., Arechavaleta A., Irigaray B., Callejas N., Grompone M., Vieitez I. (2017). Chemical and Sensory Profiles of Commercial Goat Cheeses. Int. Dairy J..

[30] Ianni A., Bennato F., Martino C., Grotta L., Martino G. (2020). Volatile Flavor Compounds in Cheese as Affected by Ruminant Diet. Molecules.

[31] D’Angelo S., Motti M.L., Meccariello R. (2020). ω-3 and ω-6 Polyunsaturated Fatty Acids, Obesity and Cancer. Nutrients.

[32] Simopoulos A.P. (2002). The Importance of the Ratio of Omega-6/Omega-3 Essential Fatty Acids. Biomed. Pharmacother..

[33] Cossignani L., Giua L., Urbani E., Simonetti M.S., Blasi F. (2014). Fatty Acid Composition and CLA Content in Goat Milk and Cheese Samples from Umbrian Market. Eur. Food Res. Technol..

[34] Kawęcka A., Radkowska I., Kawęcka A., Sikora J. (2020). Concentratıons of Selected Bıoactıve Components in Tradıtıonal Cheeses Made from Goat’s, Cow’s and Sheep’s Mılk. J. Elem..

[35] Department of Health Nutritional Aspects of Cardiovascular Disease (1994). Report of the Cardiovascular Review Group Committee on Medical Aspects of Food Policy. Rep. Health Soc. Subj..

[36] Galina M.A., Pineda J., Piedrahita R.I.H., Vazquez P., Haenlein G., Olmos J., Park Y.W. (2019). Effect of Grazing on the Fatty Acid Composition of Goat’s Milk or Cheese. Adv. Dairy Res..

[37] Yurchenko S., Sats A., Tatar V., Kaart T., Mootse H., Jõudu I. (2018). Fatty Acid Profile of Milk from Saanen and Swedish Landrace Goats. Food Chem..

[38] Pilarczyk R., Wójcik J. (2015). Fatty Acids Profile and Health Lipid Indices in the Longissimus Lumborum Muscle of Different Beef Cattle Breeds Reared under Intensive Production Systems. Acta Sci. Pol. Zootech..

[39] Dimitrova C., Stoycheva S., Ivanova S. (2020). Fatty Acid Profile and Qualitative Evaluation of the Fat Fraction in Goat White Brined Cheese on the 45th Day of the Ripening Process. Ser. D. Anim. Sci..

[40] Jensen R.G. (2002). The Composition of Bovine Milk Lipids: January 1995 to December 2000. J. Dairy Sci..

[41] Chilliard Y., Ferlay A., Rouel J., Lamberet G. (2003). A Review of Nutritional and Physiological Factors Affecting Goat Milk Lipid Synthesis and Lipolysis. J. Dairy Sci..

[42] Narducci V., Finotti E., Galli V., Carcea M. (2019). Lipids and Fatty Acids in Italian Durum Wheat (Triticum Durum Desf.) Cultivars. Foods.

[43] Serrapica F., Masucci F., Di Francia A., Napolitano F., Braghieri A., Esposito G., Romano R. (2020). Seasonal Variation of Chemical Composition, Fatty Acid Profile, and Sensory Properties of a Mountain Pecorino Cheese. Foods.

[44] Coulon J.-B., Delacroix-Buchet A., Martin B., Pirisi A. (2004). Relationships between Ruminant Management and Sensory Characteristics of Cheeses: A Review. Lait.

[45] Salvador A., Igual M., Contreras C., Martínez-Navarrete N., del Mar Camacho M. (2014). Effect of the Inclusion of Citrus Pulp in the Diet of Goats on Cheeses Characteristics. Small Rumin. Res..

[46] Gentili A., Caretti F., Bellante S., Ventura S., Canepari S., Curini R. (2013). Comprehensive Profiling of Carotenoids and Fat-Soluble Vitamins in Milk from Different Animal Species by LC-DAD-MS/MS Hyphenation. J. Agric. Food Chem..

[47] de Medeiros E.J.L., do Egypto Queiroga R.d.C.R., de Medeiros A.N., Bomfim M.A.D., Batista A.S.M., dos Santos Félex S.S., Madruga M.S. (2013). Sensory Profile and Physicochemical Parameters of Cheese from Dairy Goats Fed Vegetable Oils in the Semiarid Region of Brazil. Small Rumin. Res..

[48] Milovanovic B., Djekic I., Miocinovic J., Djordjevic V., Lorenzo J.M., Barba F.J., Mörlein D., Tomasevic I. (2020). What Is the Color of Milk and Dairy Products and How Is It Measured?. Foods.

[49] Park Y.W. (2007). Rheological Characteristics of Goat and Sheep Milk. Small Rumin. Res..

[50] Álvarez S., Fresno M., Méndez P., Castro N., Fernández J.R., Sanz Sampelayo M.R. (2007). Alternatives for Improving Physical, Chemical, and Sensory Characteristics of Goat Cheeses: The Use of Arid-Land Forages in the Diet. J. Dairy Sci..

[51] Baysal S., Ozcan T. (2020). Characterisation and Consumer Liking of White Cheeses from Different Milk Fermentation: Correlation between Sensorial and Instrumental Analyses. Int. Food Res. J..

[52] Fontecha J., Peláez C., Juárez M., Requena T., Gómez C., Ramos M. (1990). Biochemical and Microbiological Characteristics of Artisanal Hard Goat’s Cheese. J. Dairy Sci..

[53] Bintsis T. (2018). Lactic Acid Bacteria as Starter Cultures: An Update in Their Metabolism and Genetics. AIMS Microbiol..

[54] Coelho M.C., Malcata F.X., Silva C.C.G. (2022). Lactic Acid Bacteria in Raw-Milk Cheeses: From Starter Cultures to Probiotic Functions. Foods.

[55] El Zubeir I.E.M., Ahmed M.I.A. (2007). The Hygienic Quality of Raw Milk Produced by Some Dairy Farms in Khartoum State, Sudan. Res. J. Microbiol..

[56] Psoni L., Tzanetakis N., Litopoulou-Tzanetaki E. (2003). Microbiological Characteristics of Batzos, a Traditional Greek Cheese from Raw Goat’s Milk. Food Microbiol..

[57] Kawęcka A., Pasternak M. (2024). Chemical, Nutritional and Sensory Characteristics of Milk and Cheeses Obtained from Autochthonous, Cosmopolitan, and Crossbred Goats. Ann. Anim. Sci..

[58] Iriondo-DeHond M., Blázquez-Duff J.M., del Castillo M.D., Miguel E. (2020). Nutritional Quality, Sensory Analysis and Shelf Life Stability of Yogurts Containing Inulin-Type Fructans and Winery Byproducts for Sustainable Health. Foods.

[59] Cliff M.A., Fan L., Sanford K., Stanich K., Doucette C., Raymond N. (2013). Descriptive Analysis and Early-Stage Consumer Acceptance of Yogurts Fermented with Carrot Juice. J. Dairy Sci..

[60] Drub T., dos Santos F., Centeno A., Capriles V. (2021). Sorghum, Millet and Pseudocereals as Ingredients for Gluten-Free Whole-Grain Yeast Rolls. Int. J. Gastron. Food Sci..

[61] Herrera T., Iriondo-DeHond M., Ramos Sanz A., Bautista A.I., Miguel E. (2023). Effect of Wild Strawberry Tree and Hawthorn Extracts Fortification on Functional, Physicochemical, Microbiological, and Sensory Properties of Yogurt. Foods.

[62] Poveda J.M., Cabezas L. (2006). Free Fatty Acid Composition of Regionally-Produced Spanish Goat Cheese and Relationship with Sensory Characteristics. Food Chem..

[63] Teng D., Wilcock A., Aung M. (2004). Cheese Quality at Farmers Markets: Observation of Vendor Practices and Survey of Consumer Perceptions. Food Control.

