# Supplementary material for: Physico-Chemical, Microbiological and Sensory Characteristics of Cabra del Guadarrama Cheese and Other Cheeses from Different Spanish Autochthonous Goat Breeds
Source: Foods. 2025 Jul 3;14(13):2368. doi: 10.3390/foods14132368 (PMC12248980; doi:10.3390/foods14132368)
Supplement: Supplementary file 1 [file foods-14-02368-s001.zip › foods-3704625-supplementary.pdf]

Table S1. Correlations coefficients between physico-chemical, color and texture instrumental variables

Variables	Fat	Prot	pH	Moist	a_w	Salt	*L** paste	*a** paste	*b** paste	*L** rind	*a** rind	*b** rind	Hard 50	Spring 50	Cohe 50	Adh 50	Chew 50	Res 50	Gumm 50	Hard 75	Spring 75	Cohe 75	Adh 75	Chew 75	Res 75	Gumm 75
**Fat**	1																									
**Prot**	-0.422	1																								
**pH**	-0.686	0.553	**1**																							
**Moist**	-0.772	-0.203	0.192	1																						
**a_w**	-0.842	-0.035	0.236	**0.983**	**1**																					
**Salt**	0.931	-0.658	-0.578	-0.604	-0.730	1																				
***L** paste**	0.421	-0.825	-0.869	0.233	0.134	0.478	1																			
***a** paste**	-0.283	-0.080	-0.492	0.582	0.636	-0.403	0.560	1																		
***b** paste**	-0.332	0.801	0.839	-0.320	-0.229	-0.391	**-0.995**	-0.626	1																	
***L** rind**	0.560	-0.660	-0.063	-0.332	-0.493	0.807	0.186	-0.695	-0.116	1																
***a** rind**	0.493	0.183	-0.717	-0.442	-0.356	0.169	0.322	0.471	-0.302	-0.442	1															
***b** rind**	0.802	-0.110	-0.838	-0.630	-0.609	0.555	0.460	0.223	-0.406	-0.039	0.913	1														
**Hard50**	0.863	0.066	-0.341	**-0.987**	**-0.987**	0.703	-0.071	-0.508	0.162	0.378	0.502	0.720	1													
**Spring50**	-0.433	0.544	-0.127	0.306	0.456	-0.696	-0.014	0.790	-0.051	**-0.982**	0.569	0.191	-0.323	1												
**Cohe50**	-0.942	0.309	0.404	0.866	0.940	-0.919	-0.147	0.587	0.049	-0.701	-0.266	-0.608	-0.915	0.628	1											
**Adh50**	-0.882	-0.021	0.357	**0.980**	**0.989**	-0.735	0.041	0.515	-0.133	-0.416	-0.485	-0.719	**-0.999**	0.356	0.932	1										
**Chew50**	0.082	0.861	0.141	-0.605	-0.450	-0.241	-0.595	-0.114	0.612	-0.508	0.568	0.388	0.516	0.467	-0.131	-0.476	1									
**Res50**	-0.905	0.443	0.359	0.765	0.870	**-0.954**	-0.193	0.634	0.097	-0.826	-0.099	-0.481	-0.821	0.759	**0.980**	0.845	0.049	1								
**Gumm50**	0.291	0.732	0.183	-0.816	-0.704	0.032	-0.642	-0.442	0.688	-0.165	0.427	0.383	0.727	0.117	-0.419	-0.696	0.933	-0.269	1							
**Hard75**	0.500	0.543	0.060	-0.932	-0.857	0.276	-0.519	-0.550	0.585	0.057	0.423	0.480	0.868	-0.081	-0.630	-0.846	0.829	-0.498	**0.969**	**1**						
**Spring75**	-0.470	0.658	-0.013	0.249	0.412	-0.743	-0.155	0.697	0.092	**-0.994**	0.533	0.142	-0.288	**0.990**	0.624	0.326	0.559	0.763	0.222	0.012	1					
**Cohe75**	**-0.955**	0.507	0.505	0.740	0.844	**-0.982**	-0.330	0.502	0.235	-0.776	-0.214	-0.588	-0.817	0.681	**0.976**	0.843	0.072	**0.987**	-0.215	-0.449	0.705	1				
**Adh75**	-0.644	-0.401	0.105	**0.977**	0.921	-0.423	0.361	0.515	-0.436	-0.138	-0.501	-0.606	-0.940	0.130	0.738	0.924	-0.762	0.609	-0.918	**-0.984**	0.057	0.580	1			
**Chew75**	0.213	0.633	-0.291	-0.488	-0.340	-0.157	-0.164	0.290	0.173	-0.639	0.875	0.678	0.467	0.681	-0.099	-0.432	0.881	0.098	0.722	0.634	0.711	0.042	-0.628	1		
**Res75**	-0.446	-0.006	-0.336	0.688	0.750	-0.543	0.451	**0.985**	-0.529	-0.745	0.344	0.058	-0.635	0.811	0.720	0.645	-0.131	0.755	-0.474	-0.611	0.729	0.640	0.604	0.224	1	
**Gumm75**	-0.071	0.930	0.404	-0.550	-0.400	-0.334	-0.795	-0.292	0.807	-0.434	0.317	0.139	0.427	0.345	-0.063	-0.387	**0.961**	0.089	0.931	0.814	0.463	0.153	-0.710	0.720	-0.268	1

Coefficients in bold indicate a statistically significant correlation ($p<0.05$). Nutritional: Prot= protein. Physico-chemical: Moist = moisture. Color (paste and rind): *L**= lightness, *a**= red index, *b**= yelow index. Texture instrumental variables at 50% and 75 % compression (50 and 75, respectively): Hard50 and Hard75= hardness, Spring50 and Spring75= springiness, Cohe50 and Cohe75 = cohesiveness, Adh50 and Adh75 = adhesiveness, Chew50 and Chew75 = chewiness, Res50 and Res75 = resilience, Gumm50 and Gumm75 = gumminess.

Table S2. Correlations coefficients between nutritional, physico-chemical, color, microorganism counts, sum of fatty acids profile and health lipid indices variables.

Variables	Fat	Prot	pH	Moist	a_w	Salt	L* paste	a* paste	b* paste	L* rind	a* rind	b* rind	Lactob	Lactoc	MAB	Mold	Yeast	HLB	Entero	SCFA	SUFA	MUFA	PUFA	UFA	n6/n3	AI	TI	DFA	OFA	H/H
**Fat**	1																													
**Prot**	-0.422	1																												
**pH**	-0.686	0.553	1																											
**Moist**	-0.772	-0.203	0.192	1																										
**a_w**	-0.842	-0.035	0.236	**0.983**	1																									
**Salt**	0.931	-0.658	-0.578	-0.604	-0.730	1																								
**L* paste**	0.421	-0.825	-0.869	0.233	0.134	0.478	1																							
**a* paste**	-0.283	-0.080	-0.492	0.582	0.636	-0.403	0.560	1																						
**b* paste**	-0.332	0.801	0.839	-0.320	-0.229	-0.391	**-0.995**	-0.626	1																					
**L* rind**	0.560	-0.660	-0.063	-0.332	-0.493	0.807	0.186	-0.695	-0.116	1																				
**a* rind**	0.493	0.183	-0.717	-0.442	-0.356	0.169	0.322	0.471	-0.302	-0.442	1																			
**b* rind**	0.802	-0.110	-0.838	-0.630	-0.609	0.555	0.460	0.223	-0.406	-0.039	0.913	1																		
**Lactob**	-0.695	0.379	**0.980**	0.302	0.313	-0.519	-0.759	-0.489	0.726	0.055	-0.841	-0.919	1																	
**Lactoc**	-0.820	0.227	0.896	0.570	0.567	-0.604	-0.563	-0.258	0.510	-0.017	-0.882	**-0.992**	**0.954**	1																
**MAB**	-0.738	0.167	0.897	0.497	0.477	-0.497	-0.564	-0.369	0.522	0.111	-0.928	**-0.987**	**0.965**	**0.991**	1															
**Mold**	-0.887	0.557	0.389	0.672	0.795	**-0.975**	-0.284	0.595	0.191	-0.875	-0.036	-0.435	0.339	0.469	0.349	1														
**Yeast**	-0.887	0.557	0.389	0.672	0.795	**-0.975**	-0.284	0.595	0.191	-0.875	-0.036	-0.435	0.339	0.469	0.349	**1.000**	1													
**HLB**	-0.208	0.575	0.844	-0.359	-0.321	-0.172	-0.928	-0.822	**0.950**	0.192	-0.489	-0.474	0.778	0.555	0.604	-0.048	-0.048	1												
**Entero**	0.255	0.567	0.453	-0.787	-0.735	0.155	-0.770	-0.812	0.828	0.218	-0.020	0.058	0.333	0.037	0.098	-0.333	-0.333	0.851	1											
**SCFA**	0.745	-0.398	**-0.978**	-0.355	-0.374	0.576	0.749	0.426	-0.710	0.008	0.827	0.932	**-0.997**	**-0.967**	**-0.968**	-0.404	-0.404	-0.744	-0.286	1										
**SUFA**	0.938	-0.492	-0.894	-0.580	-0.638	0.833	0.655	0.065	-0.585	0.350	0.666	0.904	-0.897	-0.939	-0.891	-0.716	-0.716	-0.521	-0.043	0.926	1									
**MUFA**	0.732	-0.660	-0.241	-0.481	-0.632	0.917	0.271	-0.645	-0.189	**0.974**	-0.229	0.187	-0.146	-0.238	-0.111	**-0.960**	**-0.960**	0.098	0.245	0.211	0.544	1								
**PUFA**	**0.977**	-0.231	-0.559	-0.891	-0.934	0.869	0.224	-0.395	-0.130	0.508	0.507	0.790	-0.603	-0.783	-0.700	-0.859	-0.859	-0.031	0.447	0.657	0.869	0.683	1							
**UFA**	0.875	-0.561	-0.371	-0.662	-0.787	**0.972**	0.275	-0.608	-0.183	0.887	0.012	0.413	-0.318	-0.448	-0.326	**-1.000**	**-1.000**	0.061	0.335	0.384	0.700	**0.967**	0.847	1						
**n6/n3**	0.168	-0.078	-0.775	0.123	0.181	-0.056	0.628	0.874	-0.652	-0.577	0.824	0.667	-0.820	-0.686	-0.770	0.260	0.260	-0.845	-0.571	0.777	0.483	-0.426	0.084	-0.280	1					
**AI**	0.337	-0.133	-0.856	-0.028	0.018	0.105	0.662	0.781	-0.669	-0.460	0.882	0.782	-0.904	-0.802	-0.868	0.095	0.095	-0.835	-0.488	0.872	0.625	-0.285	0.256	-0.117	**0.985**	1				
**TI**	0.918	-0.464	-0.396	-0.758	-0.863	**0.964**	0.229	-0.603	-0.133	0.818	0.124	0.506	-0.370	-0.527	-0.410	**-0.992**	**-0.992**	0.081	0.411	0.436	0.743	0.922	0.911	**0.990**	-0.227	-0.056	1			
**DFA**	0.894	-0.517	-0.939	-0.493	-0.549	0.785	0.722	0.175	-0.662	0.284	0.693	0.906	-0.936	-0.947	-0.911	-0.648	-0.648	-0.614	-0.148	**0.959**	**0.994**	0.478	0.808	0.631	0.567	0.697	0.670	1		
**OFA**	-0.099	0.139	0.759	-0.236	-0.284	0.093	-0.672	-0.919	0.704	0.572	-0.749	-0.587	0.784	0.617	0.705	-0.303	-0.303	0.887	0.666	-0.737	-0.430	0.440	0.004	0.322	**-0.993**	**-0.964**	0.284	-0.522	1	
**H/H**	0.710	-0.532	**-0.999**	-0.233	-0.275	0.593	0.849	0.468	-0.815	0.068	0.735	0.860	**-0.985**	-0.914	-0.913	-0.408	-0.408	-0.822	-0.415	**0.986**	0.909	0.252	0.589	0.390	0.768	0.854	0.419	**0.951**	-0.747	1

Coefficients in bold and grey shading indicate a statistically significant correlation ($p<0.05$). Coefficients in bold and blue shading indicate a statistically significant correlation ($p<0.01$). Nutritional: Prot= protein. Physicio-chemical: Moist = moisture. Color (paste and rind): *L**= lightness; *a**= red index; *b**= yelow index. Microorganisms counts: Lactob = lactobacilli, Lactoc = lactococc, MAB = mesophilic aerobic bacteria, HLB = heterofermentative lactic bacteria, Entero = enterobacteriaceae. Sum of fatty acids profile and health lipid indices: SCFA = all short-chain fatty acids (C4:0, C6:0, C8:0, C10:0), SUFA = all saturated fatty acids, MUFA = all monounsaturated fatty acids, PUFA = all polyunsaturated fatty acids, UFA = all unsaturated fatty acids (Σ MUFA + Σ PUFA), n6/n3 ratio = Σ n6 / Σ n3, AI = Index of Atherogenicity, TI =Index of Thrombogenicity, DFA = Hypocholesterolemic fatty acids (Σ UFA + C18:0), OFA = Hypercholesterolemic fatty acids (Σ SUFA-C18:0), H/H = hypocholesterolemic/hypercholesterolemic ratio.

Table S3. Correlations coefficients between texture instruments, sum of fatty acids profile and health lipid indices variables.

	Hard 50	Spring 50	Cohe 50	Adh 50	Chew 50	Res 50	Gumm 50	Hard 75	Spring 75	Cohe 75	Adh 75	Chew 75	Res 75	Gumm 75	SCFA	SUFA	MUFA	PUFA	UFA	n6/n3	AI	TI	DFA	OFA	H/H
**Hard50**	1																								
**Spring50**	-0.323	1																							
**Cohe50**	-0.915	0.628	1																						
**Adh50**	**-0.999**	0.356	0.932	1																					
**Chew50**	0.516	0.467	-0.131	-0.476	1																				
**Res50**	-0.821	0.759	**0.980**	0.845	0.049	1																			
**Gumm50**	0.727	0.117	-0.419	-0.696	0.933	-0.269	1																		
**Hard75**	0.868	-0.081	-0.630	-0.846	0.829	-0.498	**0.969**	1																	
**Spring75**	-0.288	**0.990**	0.624	0.326	0.559	0.763	0.222	0.012	1																
**Cohe75**	-0.817	0.681	**0.976**	0.843	0.072	**0.987**	-0.215	-0.449	0.705	1															
**Adh75**	-0.940	0.130	0.738	0.924	-0.762	0.609	-0.918	**-0.984**	0.057	0.580	1														
**Chew75**	0.467	0.681	-0.099	-0.432	0.881	0.098	0.722	0.634	0.711	0.042	-0.628	1													
**Res75**	-0.635	0.811	0.720	0.645	-0.131	0.755	-0.474	-0.611	0.729	0.640	0.604	0.224	1												
**Gumm75**	0.427	0.345	-0.063	-0.387	**0.961**	0.089	0.931	0.814	0.463	0.153	-0.710	0.720	-0.268	1											
**SCFA**	0.488	0.175	-0.482	-0.497	0.059	-0.402	0.023	0.137	0.081	-0.540	-0.292	0.458	0.261	-0.210	1										
**SUFA**	0.704	-0.182	-0.769	-0.722	0.017	-0.717	0.120	0.301	-0.257	-0.817	-0.466	0.302	-0.110	-0.204	0.926	1									
**MUFA**	0.544	-0.924	-0.831	-0.580	-0.398	-0.924	-0.060	0.180	-0.945	-0.897	-0.286	-0.468	-0.731	-0.379	0.211	0.544	1								
**PUFA**	**0.952**	-0.408	**-0.965**	**-0.963**	0.266	-0.904	0.486	0.674	-0.416	-0.931	-0.792	0.323	-0.548	0.137	0.657	0.869	0.683	1							
**UFA**	0.730	-0.815	-0.943	-0.760	-0.196	**-0.989**	0.127	0.367	-0.833	-.979*	-0.486	-0.227	-0.724	-0.228	0.384	0.700	**0.967**	0.847	1						
**n6/n3**	-0.026	0.721	0.158	0.034	0.140	0.262	-0.119	-0.160	0.630	0.109	0.076	0.586	0.783	-0.118	0.777	0.483	-0.426	0.084	-0.280	1					
**AI**	0.135	0.619	-0.017	-0.130	0.164	0.092	-0.047	-0.051	0.527	-0.062	-0.054	0.610	0.666	-0.109	0.872	0.625	-0.285	0.256	-0.117	**0.985**	1				
**TI**	0.819	-0.744	**-0.981**	-0.844	-0.060	**-0.999**	0.252	0.483	-0.754	**-0.993**	-0.600	-0.089	-0.728	-0.109	0.436	0.743	0.922	0.911	**0.990**	-0.227	-0.056	1			
**DFA**	0.626	-0.107	-0.692	-0.644	-0.022	-0.641	0.046	0.216	-0.193	-0.755	-0.385	0.306	0.001	-0.259	**0.959**	**0.994**	0.478	0.808	0.631	0.567	0.697	0.670	1		
**OFA**	0.134	-0.714	-0.234	-0.140	-0.035	-0.320	0.235	0.279	-0.614	-0.165	-0.196	-0.493	-0.839	0.213	-0.737	-0.430	0.440	0.004	0.322	**-0.993**	**-0.964**	0.284	-0.522	1	
**H/H**	0.379	0.121	-0.434	-0.395	-0.107	-0.383	-0.143	-0.017	0.011	-0.527	-0.148	0.317	0.308	-0.371	**0.986**	0.909	0.252	0.589	0.390	0.768	0.854	0.419	**0.951**	-0.747	1

Coefficients in bold and grey shading indicate a statistically significant correlation ($p<0.05$). Coefficients in bold and blue shading indicate a statistically significant correlation ($p<0.01$). Texture instrumental variables at 50% and 75 % compression (50 and 75, respectively): Hard50 and Hard75= hardness, Spring50 and Spring75= springiness, Cohe50 and Cohe75 = cohesiveness, Adh50 and Adh75 = adhesiveness, Chew50 and Chew75 = chewiness, Res50 and Res75 = resilience, Gumm50 and Gumm75 = gumminess. Sum of fatty acids profile and health lipid indices: SCFA = all short-chain fatty acids (C4:0, C6:0, C8:0, C10:0), SUFA = all saturated fatty acids, MUFA = all monounsaturated fatty acids, PUFA = all polyunsaturated fatty acids, UFA = all unsaturated fatty acids (Σ MUFA + Σ PUFA), n6/n3 ratio = Σ n6 / Σ n3, AI = Index of Atherogenicity, TI =Index of Thrombogenicity, DFA = Hypocholesterolemic fatty acids (Σ UFA + C18:0), OFA = Hypercholesterolemic fatty acids (Σ SUFA-C18:0), H/H = hypocholesterolemic/hypercholesterolemic ratio.

Table S4. Correlations coefficients between nutritional, physico-chemical, color and sensorial variables.

Variables	Fat	Prot	pH	Moist	a_w	Salt	*L** paste	*a** paste	*b** paste	*L** rind	*a** rind	*b** rind	Appe BC	Smell BC	Flavor BC	TexT BC	OL BC	Appe IC	Smell IC	Flavor IC	Text IC	OL IC	Spring	SR	SH	Firm	Friab	Adhe	Juic	AF	SF	BF	SWF	OP	OQ	NC
**Fat**	1																																			
**Prot**	-0.422	1																																		
**pH**	-0.686	0.553	1																																	
**Moist**	-0.772	-0.203	0.192	1																																
**a_w**	-0.842	-0.035	0.236	**0.983**	1																															
**Salt**	0.931	-0.658	-0.578	-0.604	-0.730	1																														
***L** paste**	0.421	-0.825	-0.869	0.233	0.134	0.478	1																													
***a** paste**	-0.283	-0.080	-0.492	0.582	0.636	-0.403	0.560	1																												
***b** paste**	-0.332	0.801	0.839	-0.320	-0.229	-0.391	**-0.995**	-0.626	1																											
***L** rind**	0.560	-0.660	-0.063	-0.332	-0.493	0.807	0.186	-0.695	-0.116	1																										
***a** rind**	0.493	0.183	-0.717	-0.442	-0.356	0.169	0.322	0.471	-0.302	-0.442	1																									
***b** rind**	0.802	-0.110	-0.838	-0.630	-0.609	0.555	0.460	0.223	-0.406	-0.039	0.913	1																								
**Appe BC**	0.678	0.002	-0.815	-0.542	-0.493	0.394	0.430	0.357	-0.391	-0.222	**0.972**	**0.983**	1																							
**Smell BC**	0.443	0.071	-0.788	-0.294	-0.220	0.144	0.459	0.605	-0.449	-0.467	**0.984**	0.887	**0.954**	1																						
**Flavor BC**	-0.830	0.487	0.255	0.689	0.809	-0.934	-0.156	0.703	0.063	-0.902	0.061	-0.337	-0.159	0.131	1																					
**Text BC**	-0.644	-0.398	0.358	0.879	0.797	-0.342	0.147	0.162	-0.208	0.118	-0.785	-0.809	-0.802	-0.663	0.319	1																				
**OL BC**	-0.728	-0.312	0.271	**0.973**	0.922	-0.489	0.207	0.398	-0.283	-0.119	-0.620	-0.733	-0.681	-0.480	0.526	**0.966**	1																			
**Appe IC**	0.631	-0.736	**-0.969**	-0.039	-0.121	0.612	**0.959**	0.475	-0.933	0.199	0.524	0.688	0.646	0.623	-0.291	-0.137	-0.079	1																		
**Smell IC**	0.501	-0.545	**-0.973**	0.026	-0.009	0.402	0.912	0.671	-0.904	-0.084	0.680	0.733	0.742	0.783	-0.050	-0.204	-0.078	**0.959**	1																	
**Flavor IC**	**-0.982**	0.248	0.592	0.876	0.920	-0.870	-0.258	0.358	0.166	-0.493	-0.532	-0.811	-0.696	-0.452	0.810	0.761	0.843	-0.499	-0.391	1																
**Text IC**	-0.722	-0.221	0.578	0.801	0.736	-0.421	-0.100	-0.020	0.042	0.125	-0.891	-0.929	-0.924	-0.805	0.310	**0.968**	0.906	-0.373	-0.443	0.800	1															
**OL IC**	-0.703	-0.315	0.436	0.880	0.810	-0.408	0.061	0.138	-0.125	0.075	-0.807	-0.855	-0.840	-0.694	0.362	**0.996**	.963*	-0.223	-0.279	0.805	**0.984**	1														
**Spring**	**-0.993**	0.518	0.736	0.695	0.779	-0.950	-0.515	0.226	0.429	-0.584	-0.469	-0.788	-0.661	-0.438	0.823	0.566	0.648	-0.702	-0.564	**0.954**	0.667	0.632	1													
**SR**	-0.340	0.689	0.890	-0.271	-0.207	-0.334	**-0.977**	-0.711	**0.984**	0.024	-0.454	-0.512	-0.518	-0.594	-0.016	-0.084	-0.196	-0.944	**-0.958**	0.192	0.168	-0.005	0.428	1												
**SH**	-0.306	-0.505	-0.423	0.808	0.769	-0.222	0.732	0.843	-0.795	-0.286	0.034	-0.074	-0.003	0.213	0.496	0.589	0.731	0.542	0.610	0.457	0.385	0.544	0.206	-0.783	1											
**Firm**	0.777	0.189	-0.191	-1.000**	-.986*	0.615	-0.229	-0.593	0.317	0.350	0.429	0.624	0.532	0.281	-0.702	-0.871	-.968*	0.041	-0.029	-0.880	-0.792	-0.872	-0.701	0.271	-0.809	1										
**Friab**	0.942	-0.216	-0.768	-0.776	-0.791	0.759	0.405	-0.058	-0.328	0.248	0.756	**0.952**	0.882	0.705	-0.606	-0.808	-0.811	0.646	0.610	**-0.950**	-0.898	-0.858	-0.923	-0.395	-0.254	0.774	1									
**Adhe**	0.085	-0.806	0.043	0.298	0.124	0.441	0.350	-0.324	-0.334	0.801	-0.718	-0.428	-0.558	-0.647	-0.478	0.675	0.495	0.192	-0.053	0.051	0.628	0.630	-0.160	-0.163	0.232	-0.280	-0.231	1								
**Juic**	-0.876	-0.001	0.310	**0.979**	**0.997**	-0.753	0.067	0.578	-0.161	-0.476	-0.414	-0.666	-0.553	-0.286	0.804	0.816	0.929	-0.192	-0.086	0.946	0.772	0.833	0.817	-0.134	0.720	**-0.982**	-0.837	0.138	1							
**AF**	0.104	-0.295	-0.789	0.322	0.341	-0.031	0.776	0.923	-0.808	-0.466	0.654	0.531	0.616	0.779	0.385	-0.050	0.156	0.761	0.902	-0.011	-0.274	-0.101	-0.166	-0.892	0.778	-0.330	0.301	-0.255	0.268	1						
**SF**	0.517	0.193	-0.707	-0.481	-0.395	0.190	0.299	0.431	-0.275	-0.419	**0.999**	0.922	**0.976**	**0.975**	0.029	-0.811	-0.654	0.510	0.660	-0.560	-0.910	-0.833	-0.488	-0.427	-0.008	0.468	0.774	-0.720	-0.451	0.622	1					
**BF**	-0.214	0.717	-0.113	-0.064	0.107	-0.552	-0.198	0.553	0.162	-0.915	0.714	0.376	0.532	0.688	0.652	-0.508	-0.286	-0.094	0.179	0.112	-0.506	-0.471	0.265	-0.010	0.027	0.045	0.124	**-0.965**	0.083	0.446	0.704	1				
**SWF**	0.021	-0.893	-0.149	0.497	0.330	0.352	0.580	0.001	-0.584	0.619	-0.578	-0.349	-0.443	-0.457	-0.283	0.750	0.641	0.390	0.196	0.150	0.634	0.693	-0.120	-0.433	0.533	-0.481	-0.236	0.945	0.323	0.060	-0.593	-0.832	1			
**OP**	-0.172	0.833	0.017	-0.226	-0.048	-0.518	-0.382	0.347	0.358	-0.842	0.667	0.353	0.492	0.602	0.546	-0.610	-0.425	-0.244	0.010	0.036	-0.557	-0.561	0.245	0.189	-0.201	0.207	0.146	**-0.996**	-0.059	0.244	0.667	**0.973**	-0.936	1		
**OQ**	0.382	-0.108	0.370	-0.562	-0.647	0.540	-0.398	**-0.982**	0.469	0.818	-0.483	-0.178	-0.333	-0.598	-0.804	-0.109	-0.360	-0.323	-0.553	-0.425	0.037	-0.102	-0.342	0.572	-0.753	0.575	0.122	0.463	-0.596	-0.858	-0.445	-0.677	0.155	-0.493	1	
**NC**	**0.995**	-0.511	-0.719	-0.707	-0.791	**0.953***	0.496	-0.250	-0.409	0.597	0.456	0.779	0.650	0.421	-0.835	-0.569	-0.656	0.685	0.543	**-0.958**	-0.664	-0.634	**-1.000**	-0.406	-0.228	0.713	0.920	0.164	-0.828	0.141	0.476	-0.275	0.116	-0.250	0.364	1

Coefficients in bold and grey shading indicate a statistically significant correlation ($p<0.05$). Coefficients in bold and blue shading indicate a statistically significant correlation ($p<0.01$). Nutritional: Prot= protein. Physico-chemical: Moist = moisture. Color (paste and rind): *L**= lightness, *a**= red index, *b**= yelow index. Sensorial consumers: BC = Blind condictions, IC = Informed conditions. Appe= appearance, Text= texture, OL= overall liking. Sensorial panelist: Spring = springiness, SR = surface roughness, SH = surface humidity, Firm= firmness, Friab= friability, Adhe = adherence, Juic= juiciness, AF = acid flavor, SF = salty flavor, BF = bitter flavor, SWF = sweet flavor, OP = overall persistence, OQ = overall quality, NC = number of chews.

Table S5. Correlations coefficients between sensorial, sum of fatty acids profile and health lipid indices variables.

Variables	Appe BC	Smell BC	Flavor BC	TexT BC	OL BC	Appe IC	Smell IC	Flavor IC	Text IC	OL IC	Spring	SR	SH	Firm	Friab	Adhe	Juic	AF	SF	BF	SWF	OP	OQ	NC	SCFA	SUFA	MUFA	PUFA	UFA	n6/n3	AI	TI	DFA	OFA	H/H
**Appe BC**	1																																	
**Smell BC**	**0.954**	1																																
**Flavor BC**	-0.159	0.131	1																															
**Text BC**	-0.802	-0.663	0.319	1																														
**OL BC**	-0.681	-0.480	0.526	**0.966**	1																													
**Appe IC**	0.646	0.623	-0.291	-0.137	-0.079	1																												
**Smell IC**	0.742	0.783	-0.050	-0.204	-0.078	**0.959**	1																											
**Flavor IC**	-0.696	-0.452	0.810	0.761	0.843	-0.499	-0.391	1																										
**Text IC**	-0.924	-0.805	0.310	**0.968**	0.906	-0.373	-0.443	0.800	1																									
**OL IC**	-0.840	-0.694	0.362	**0.996**	.963*	-0.223	-0.279	0.805	**0.984**	1																								
**Spring**	-0.661	-0.438	0.823	0.566	0.648	-0.702	-0.564	**0.954**	0.667	0.632	1																							
**SR**	-0.518	-0.594	-0.016	-0.084	-0.196	-0.944	**-0.958**	0.192	0.168	-0.005	0.428	1																						
**SH**	-0.003	0.213	0.496	0.589	0.731	0.542	0.610	0.457	0.385	0.544	0.206	-0.783	1																					
**Firm**	0.532	0.281	-0.702	-0.871	**-0.968**	0.041	-0.029	-0.880	-0.792	-0.872	-0.701	0.271	-0.809	1																				
**Friab**	0.882	0.705	-0.606	-0.808	-0.811	0.646	0.610	**-0.950**	-0.898	-0.858	-0.923	-0.395	-0.254	0.774	1																			
**Adhe**	-0.558	-0.647	-0.478	0.675	0.495	0.192	-0.053	0.051	0.628	0.630	-0.160	-0.163	0.232	-0.280	-0.231	1																		
**Juic**	-0.553	-0.286	0.804	0.816	0.929	-0.192	-0.086	0.946	0.772	0.833	0.817	-0.134	0.720	**-0.982**	-0.837	0.138	1																	
**AF**	0.616	0.779	0.385	-0.050	0.156	0.761	0.902	-0.011	-0.274	-0.101	-0.166	-0.892	0.778	-0.330	0.301	-0.255	0.268	1																
**SF**	**0.976**	**0.975**	0.029	-0.811	-0.654	0.510	0.660	-0.560	-0.910	-0.833	-0.488	-0.427	-0.008	0.468	0.774	-0.720	-0.451	0.622	1															
**BF**	0.532	0.688	0.652	-0.508	-0.286	-0.094	0.179	0.112	-0.506	-0.471	0.265	-0.010	0.027	0.045	0.124	**-0.965**	0.083	0.446	0.704	1														
**SWF**	-0.443	-0.457	-0.283	0.750	0.641	0.390	0.196	0.150	0.634	0.693	-0.120	-0.433	0.533	-0.481	-0.236	0.945	0.323	0.060	-0.593	-0.832	1													
**OP**	0.492	0.602	0.546	-0.610	-0.425	-0.244	0.010	0.036	-0.557	-0.561	0.245	0.189	-0.201	0.207	0.146	**-0.996**	-0.059	0.244	0.667	**0.973**	-0.936	1												
**OQ**	-0.333	-0.598	-0.804	-0.109	-0.360	-0.323	-0.553	-0.425	0.037	-0.102	-0.342	0.572	-0.753	0.575	0.122	0.463	-0.596	-0.858	-0.445	-0.677	0.155	-0.493	1											
**NC**	0.650	0.421	-0.835	-0.569	-0.656	0.685	0.543	**-0.958**	-0.664	-0.634	**-1.000**	-0.406	-0.228	0.713	0.920	0.164	-0.828	0.141	0.476	-0.275	0.116	-0.250	0.364	1										
**SCFA**	0.914	0.865	-0.278	-0.542	-0.451	0.899	0.924	-0.687	-0.734	-0.610	-0.773	-0.786	0.262	0.352	0.862	-0.203	-0.445	0.734	0.823	0.232	-0.042	0.136	-0.330	0.758	1									
**SUFA**	0.821	0.665	-0.618	-0.591	-0.596	0.837	0.766	-0.888	-0.740	-0.662	**-0.955**	-0.620	-0.001	0.582	**0.951**	-0.002	-0.694	0.441	0.676	-0.047	0.050	-0.080	0.049	0.948	0.926	1								
**MUFA**	0.002	-0.262	**-0.967**	-0.076	-0.294	0.338	0.070	-0.672	-0.093	-0.128	-0.751	-0.076	-0.315	0.497	0.460	0.683	-0.628	-0.352	-0.204	-0.811	0.517	-0.740	0.774	0.761	0.211	0.544	1							
**PUFA**	0.673	0.422	-0.824	-0.761	-0.851	0.466	0.353	**-0.999**	-0.791	-0.803	-0.946	-0.154	-0.491	0.894	0.938	-0.045	**-0.957**	-0.030	0.537	-0.127	-0.156	-0.042	0.459	**0.951**	0.657	0.869	0.683	1						
**UFA**	0.238	-0.043	**-0.992**	-0.323	-0.513	0.409	0.175	-0.839	-0.345	-0.375	-0.878	-0.110	-0.401	0.675	0.664	0.481	-0.792	-0.267	0.040	-0.634	0.321	-0.553	0.724	0.887	0.384	0.700	**0.967**	0.847	1					
**n6/n3**	0.765	0.910	0.389	-0.296	-0.073	0.680	0.859	-0.122	-0.489	-0.334	-0.201	-0.774	0.589	-0.135	0.424	-0.494	0.109	**0.961**	0.799	0.639	-0.208	0.475	-0.845	0.179	0.777	0.483	-0.426	0.084	-0.280	1				
**AI**	0.856	**0.952**	0.227	-0.409	-0.210	0.752	0.903	-0.293	-0.603	-0.454	-0.365	-0.789	0.497	0.018	0.574	-0.471	-0.056	0.932	0.862	0.584	-0.211	0.437	-0.739	0.344	0.872	0.625	-0.285	0.256	-0.117	**0.985**	1			
**TI**	0.339	0.054	**-0.983**	-0.454	-0.629	0.398	0.187	-0.902	-0.468	-0.502	-0.908	-0.082	-0.473	0.769	0.743	0.351	-0.870	-0.251	0.155	-0.522	0.187	-0.428	0.703	0.918	0.436	0.743	0.922	0.911	**0.990**	-0.227	-0.056	1		
**DFA**	0.836	0.711	-0.538	-0.544	-0.526	0.887	0.835	-0.831	-0.714	-0.618	-0.919	-0.701	0.106	0.495	0.924	-0.013	-0.611	0.539	0.698	-0.007	0.075	-0.065	-0.057	0.909	**0.959**	**0.994**	0.478	0.808	0.631	0.567	0.697	0.670	1	
**OFA**	-0.688	-0.854	-0.433	0.178	-0.046	-0.690	-0.864	0.035	0.381	0.219	0.143	0.813	-0.683	0.247	-0.339	0.416	-0.212	**-0.985**	-0.720	-0.587	0.113	-0.404	0.881	-0.119	-0.737	-0.430	0.440	0.004	0.322	**-0.993**	**-0.964**	0.284	-0.522	1
**H/H**	0.835	0.799	-0.276	-0.396	-0.312	**0.960**	**0.964**	-0.622	-0.611	-0.473	-0.755	-0.870	0.385	0.231	0.795	-0.064	-0.348	0.771	0.726	0.123	0.119	0.002	-0.349	0.739	**0.986**	0.909	0.252	0.589	0.390	0.768	0.854	0.419	**0.951**	-0.747	1

Coefficients in bold and grey shading indicate a statistically significant correlation ($p<0.05$). Coefficients in bold and blue shading indicate a statistically significant correlation ($p<0.01$). Sensorial consumers: BC = Blind conditions, IC = Informed conditions. Appe= appearance, Text= texture, OL= overall liking. Sensorial from panelist: Spring = springiness, SR = surface roughness, SH = surface humidity, Firm= firmness, Friab= friability, Adhe = adherence, Juic= juiciness, AF = acid flavor, SF = salty flavor, BF = bitter flavor, SWF = sweet flavor, OP = overall persistence, OQ = overall quality, NC = number of chews. Sum of fatty acids profile and health lipid indices: SCFA = all short-chain fatty acids (C4:0, C6:0, C8:0, C10:0), SUFA = all saturated fatty acids, MUFA = all monounsaturated fatty acids, PUFA = all polyunsaturated fatty acids, UFA = all unsaturated fatty acids (Σ MUFA + Σ PUFA), n6/n3 ratio = Σ n6 / Σ n3, AI = Index of Atherogenicity, TI =Index of Thrombogenicity, DFA = Hypocholesterolemic fatty acids (Σ UFA + C18:0), OFA = Hypercholesterolemic fatty acids (Σ SUFA-C18:0), H/H = hypocholesterolemic/hypercholesterolemic ratio.

Table S6. Correlations coefficients between microorganism counts and sensorial variables.

Variables	Lactob	Lactoc	MAB	Mold	Yeast	HLB	Entero	Appe BC	Smell BC	Flavor BC	TexT BC	OL BC	Appe IC	Smell IC	Flavor IC	Text IC	OL IC	Spring	SR	SH	Firm	Friab	Adhe	Juic	AF	SF	BF	SWF	OP	OQ	NC
**Lactob**	1																													
**Lactoc**	**0.954**	1																												
**MAB**	**0.965**	**0.991**	1																											
**Mold**	0.339	0.469	0.349	1																										
**Yeast**	0.339	0.469	0.349	**1.000**	1																									
**HLB**	0.778	0.555	0.604	-0.048	-0.048	1																								
**Entero**	0.333	0.037	0.098	-0.333	-0.333	0.851	1																							
**Appe BC**	-0.914	**-0.967**	**-0.986**	-0.261	-0.261	-0.512	0.000	1																						
**Smell BC**	-0.887	-0.874	-0.930	0.018	0.018	-0.634	-0.200	**0.954**	1																					
**Flavor BC**	0.208	0.362	0.236	**0.990**	**0.990**	-0.186	-0.431	-0.159	0.131	1																				
**Text BC**	0.518	0.735	0.722	0.341	0.341	-0.103	-0.593	-0.802	-0.663	0.319	1																			
**OL BC**	0.410	0.662	0.617	0.527	0.527	-0.252	-0.722	-0.681	-0.480	0.526	**0.966**	1																		
**Appe IC**	-0.899	-0.771	-0.762	-0.423	-0.423	-0.882	-0.582	0.646	0.623	-0.291	-0.137	-0.079	1																	
**Smell IC**	-0.941	-0.798	-0.824	-0.192	-0.192	-0.942	-0.630	0.742	0.783	-0.050	-0.204	-0.078	**0.959**	1																
**Flavor IC**	0.635	0.806	0.726	0.851	0.851	0.071	-0.413	-0.696	-0.452	0.810	0.761	0.843	-0.499	-0.391	1															
**Text IC**	0.715	0.878	0.872	0.367	0.367	0.147	-0.383	-0.924	-0.805	0.310	**0.968**	0.906	-0.373	-0.443	0.800	1														
**OL IC**	0.584	0.790	0.773	0.394	0.394	-0.033	-0.543	-0.840	-0.694	0.362	**0.996**	.963*	-0.223	-0.279	0.805	**0.984**	1													
**Spring**	0.725	0.819	0.738	0.889	0.889	0.293	-0.151	-0.661	-0.438	0.823	0.566	0.648	-0.702	-0.564	**0.954**	0.667	0.632	1												
**SR**	0.808	0.602	0.629	0.121	0.121	**0.985**	0.807	-0.518	-0.594	-0.016	-0.084	-0.196	-0.944	**-0.958**	0.192	0.168	-0.005	0.428	1											
**SH**	-0.313	-0.015	-0.085	0.399	0.399	-0.839	**-0.997**	-0.003	0.213	0.496	0.589	0.731	0.542	0.610	0.457	0.385	0.544	0.206	-0.783	1										
**Firm**	-0.297	-0.565	-0.490	-0.685	-0.685	0.361	0.786	0.532	0.281	-0.702	-0.871	**-0.968**	0.041	-0.029	-0.880	-0.792	-0.872	-0.701	0.271	-0.809	1									
**Friab**	-0.828	-0.949	-0.904	-0.682	-0.682	-0.310	0.222	0.882	0.705	-0.606	-0.808	-0.811	0.646	0.610	**-0.950**	-0.898	-0.858	-0.923	-0.395	-0.254	0.774	1								
**Adhe**	0.232	0.332	0.415	-0.463	-0.463	-0.047	-0.288	-0.558	-0.647	-0.478	0.675	0.495	0.192	-0.053	0.051	0.628	0.630	-0.160	-0.163	0.232	-0.280	-0.231	1							
**Juic**	0.386	0.629	0.542	0.802	0.802	-0.248	-0.686	-0.553	-0.286	0.804	0.816	0.929	-0.192	-0.086	0.946	0.772	0.833	0.817	-0.134	0.720	**-0.982**	-0.837	0.138	1						
**AF**	-0.779	-0.581	-0.659	0.250	0.250	-.951*	-0.769	0.616	0.779	0.385	-0.050	0.156	0.761	0.902	-0.011	-0.274	-0.101	-0.166	-0.892	0.778	-0.330	0.301	-0.255	0.268	1					
**SF**	-0.834	-0.888	-0.930	-0.065	-0.065	-0.457	0.022	**0.976**	**0.975**	0.029	-0.811	-0.654	0.510	0.660	-0.560	-0.910	-0.833	-0.488	-0.427	-0.008	0.468	0.774	-0.720	-0.451	0.622	1				
**BF**	-0.277	-0.299	-0.405	0.616	0.616	-0.148	0.034	0.532	0.688	0.652	-0.508	-0.286	-0.094	0.179	0.112	-0.506	-0.471	0.265	-0.010	0.027	0.045	0.124	**-0.965**	0.083	0.446	0.704	1			
**SWF**	0.052	0.235	0.286	-0.307	-0.307	-0.346	-0.583	-0.443	-0.457	-0.283	0.750	0.641	0.390	0.196	0.150	0.634	0.693	-0.120	-0.433	0.533	-0.481	-0.236	0.945	0.323	0.060	-0.593	-0.832	1		
**OP**	-0.170	-0.257	-0.346	0.536	0.536	0.063	0.261	0.492	0.602	0.546	-0.610	-0.425	-0.244	0.010	0.036	-0.557	-0.561	0.245	0.189	-0.201	0.207	0.146	**-0.996**	-0.059	0.244	0.667	**0.973**	-0.936	1	
**OQ**	0.397	0.192	0.314	-0.711	-0.711	0.706	0.711	-0.333	-0.598	-0.804	-0.109	-0.360	-0.323	-0.553	-0.425	0.037	-0.102	-0.342	0.572	-0.753	0.575	0.122	0.463	-0.596	-0.858	-0.445	-0.677	0.155	-0.493	1	
**NC**	-0.708	-0.809	-0.725	-0.898	-0.898	-0.269	0.173	0.650	0.421	-0.835	-0.569	-0.656	0.685	0.543	**-0.958**	-0.664	-0.634	**-1.000**	-0.406	-0.228	0.713	0.920	0.164	-0.828	0.141	0.476	-0.275	0.116	-0.250	0.364	1

Coefficients in bold and grey shading indicate a statistically significant correlation ($p<0.05$). Coefficients in bold and blue shading indicate a statistically significant correlation ($p<0.01$). Microorganisms counts: Lactob = lactobacilli, Lactoc = lactococc, MAB = mesophilic aerobic bacteria, HLB = heterofermentative lactic bacteria, Entero = enterobacteriaceae. Sensorial consumers: BC = Blind condictions, IC = Informed conditions. Appe= appearance, Text= texture, OL= overall liking. Sensorial panelist: Spring = springiness, SR = surface roughness, SH = surface humidity, Firm= firmness, Friab= friability, Adhe = adherence, Juic= juiciness, AF = acid flavor, SF = salty flavor, BF = bitter flavor, SWF = sweet flavor, OP = overall persistence, OQ = overall quality, NC = number of chews.

Table S7. Correlations coefficients between texture instrumental and sensorial panel variables.

Variables	Hard 50	Spring 50	Cohe 50	Adh 50	Chew 50	Res 50	Gumm 50	Hard 75	Spring 75	Cohe 75	Adh 75	Chew 75	Res 75	Gumm 75	Spring	SR	SH	Firm	Friab	Adhe	Juic	AF	SF	BF	SWF	OP	OQ	NC
**Hard50**	1																											
**Spring50**	-0.323	1																										
**Cohe50**	-0.915	0.628	1																									
**Adh50**	**-0.999**	0.356	0.932	1																								
**Chew50**	0.516	0.467	-0.131	-0.476	1																							
**Res50**	-0.821	0.759	**0.980**	0.845	0.049	1																						
**Gumm50**	0.727	0.117	-0.419	-0.696	0.933	-0.269	1																					
**Hard75**	0.868	-0.081	-0.630	-0.846	0.829	-0.498	**0.969**	1																				
**Spring75**	-0.288	**0.990**	0.624	0.326	0.559	0.763	0.222	0.012	1																			
**Cohe75**	-0.817	0.681	**0.976**	0.843	0.072	**0.987**	-0.215	-0.449	0.705	1																		
**Adh75**	-0.940	0.130	0.738	0.924	-0.762	0.609	-0.918	**-0.984**	0.057	0.580	1																	
**Chew75**	0.467	0.681	-0.099	-0.432	0.881	0.098	0.722	0.634	0.711	0.042	-0.628	1																
**Res75**	-0.635	0.811	0.720	0.645	-0.131	0.755	-0.474	-0.611	0.729	0.640	0.604	0.224	1															
**Gumm75**	0.427	0.345	-0.063	-0.387	**0.961**	0.089	0.931	0.814	0.463	0.153	-0.710	0.720	-0.268	1														
**Spring**	-0.800	0.446	0.915	0.823	0.022	0.892	-0.181	-0.398	0.498	**0.952**	0.553	-0.144	0.391	0.182	1													
**SR**	0.115	-0.198	0.030	-0.093	0.469	0.044	0.586	0.505	-0.058	0.195	-0.359	0.000	-0.605	0.694	0.428	1												
**SH**	-0.707	0.375	0.564	0.692	-0.629	0.502	-0.855	-0.891	0.256	0.390	0.835	-0.260	0.847	-0.738	0.206	-0.783	1											
**Firm**	**0.987**	-0.324	-0.873	**-0.982**	0.591	-0.776	0.807	0.927	-0.268	-0.750	**-0.973**	0.471	-0.699	0.538	-0.701	0.271	-0.809	1										
**Friab**	0.862	-0.109	-0.820	-0.868	0.311	-0.724	0.419	0.572	-0.145	-0.801	-0.707	0.514	-0.228	0.105	-0.923	-0.395	-0.254	0.774	1									
**Adhe**	-0.241	-0.797	-0.164	0.199	-0.902	-0.354	-0.690	-0.537	-0.847	-0.322	0.481	**-0.953**	-0.310	-0.796	-0.160	-0.163	0.232	-0.280	-0.231	1								
**Juic**	**-0.994**	0.425	0.948	**0.997**	-0.440	0.873	-0.682	-0.840	0.391	0.860	0.914	-0.368	0.702	-0.369	0.817	-0.134	0.720	**-0.982**	-0.837	0.138	1							
**AF**	-0.204	0.617	0.235	0.202	-0.136	0.289	-0.387	-0.409	0.502	0.132	0.312	0.340	0.843	-0.378	-0.166	-0.892	0.778	-0.330	0.301	-0.255	0.268	1						
**SF**	0.538	0.544	-0.299	-0.521	0.589	-0.131	0.460	0.460	0.512	-0.242	-0.539	0.882	0.303	0.341	-0.488	-0.427	-0.008	0.468	0.774	-0.720	-0.451	0.622	1					
**BF**	0.026	0.926	0.355	0.015	0.758	0.531	0.476	0.301	0.950	0.473	-0.250	0.895	0.547	0.632	0.265	-0.010	0.027	0.045	0.124	**-0.965**	0.083	0.446	0.704	1				
**SWF**	-0.407	-0.577	0.005	0.364	**-0.991**	-0.178	-0.876	-0.746	-0.664	-0.193	0.670	-0.898	0.006	-0.947	-0.120	-0.433	0.533	-0.481	-0.236	0.945	0.323	0.060	-0.593	-0.832	1			
**OP**	0.162	0.826	0.245	-0.118	0.884	0.430	0.656	0.486	0.879	0.402	-0.418	0.924	0.346	0.792	0.245	0.189	-0.201	0.207	0.146	**-0.996**	-0.059	0.244	0.667	**0.973**	-0.936	1		
**OQ**	0.515	-0.888	-0.661	-0.531	-0.035	-0.730	0.318	0.463	-0.816	-0.612	-0.457	-0.391	**-0.985**	0.124	-0.342	0.572	-0.753	0.575	0.122	0.463	-0.596	-0.858	-0.445	-0.677	0.155	-0.493	1	
**NC**	0.810	-0.462	-0.924	-0.832	-0.016	-0.902	0.194	0.412	-0.511	**-0.958**	-0.565	0.139	-0.413	-0.171	**-1.000**	-0.406	-0.228	0.713	0.920	0.164	-0.828	0.141	0.476	-0.275	0.116	-0.250	0.364	1

Coefficients in bold and grey shading indicate a statistically significant correlation ($p<0.05$). Coefficients in bold and blue shading indicate a statistically significant correlation ($p<0.01$). Texture instrumental variables at 50% and 75 % compression (50 and 75 respectively): Hard50 and Hard75 = hardness, Spring50 and Spring75 = springiness, Cohe50 and Cohe75 = cohesiveness, Adh50 and Adh75 = adhesiveness, Chew50 and Chew75 = chewiness, Res50 and Res75 = resilience, Gumm50 and Gumm75 = gumminess. Sensorial panelist: Spring = springiness, SR = surface roughness, SH = surface humidity, Firm= firmness, Friab= friability, Adhe = adherence, Juic= juiciness, AF = acid flavor, SF = salty flavor, BF = bitter flavor, SWF = sweet flavor, OP = overall persistence, OQ = overall quality, NC = number of chews.

Table S8. Correlations coefficients between texture instrumental and sensorial consumers variables.

Variables	Hard 50	Spring 50	Cohe 50	Adh 50	Chew 50	Res 50	Gumm 50	Hard 75	Spring 75	Cohe 75	Adh 75	Chew 75	Res 75	Gumm 75	Appe BC	Smell BC	Flavor BC	TexT BC	OL BC	Appe IC	Smell IC	Flavor IC	Text IC	OL IC
**Hard50**	1																							
**Spring50**	-0.320	1																						
**Cohe50**	-0.910	0.628	1																					
**Adh50**	**-0.999**	0.356	0.932	1																				
**Chew50**	0.516	0.467	-0.130	-0.480	1																			
**Res50**	-0.820	0.759	**0.980**	0.845	0.049	1																		
**Gumm50**	0.727	0.117	-0.420	-0.700	0.933	-0.270	1																	
**Hard75**	0.868	-0.08	-0.630	-0.850	0.829	-0.500	**0.969**	1																
**Spring75**	-0.290	**0.990**	0.624	0.326	0.559	0.763	0.222	0.012	1															
**Cohe75**	-0.820	0.681	**0.976**	0.843	0.072	**0.987**	-0.220	-0.450	0.705	1														
**Adh75**	-0.940	0.13	0.738	0.924	-0.760	0.609	-0.920	**-0.984**	0.057	0.580	1													
**Chew75**	0.467	0.681	-0.100	-0.430	0.881	0.098	0.722	0.634	0.711	0.042	-0.630	1												
**Res75**	-0.630	0.811	0.720	0.645	-0.130	0.755	-0.470	-0.610	0.729	0.640	0.604	0.224	1											
**Gumm75**	0.427	0.345	-0.060	-0.390	**0.961**	0.089	0.931	0.814	0.463	0.153	-0.710	0.720	-0.270	1										
**Appe BC**	0.623	0.369	-0.46	-0.620	0.458	-0.310	0.388	0.443	0.321	-0.430	-0.550	0.772	0.205	0.201	1									
**Smell BC**	0.373	0.609	-0.17	-0.360	0.432	-0.020	0.259	0.253	0.551	-0.160	-0.340	0.801	0.480	0.166	**0.954**	1								
**Flavor BC**	-0.740	0.854	0.939	0.765	0.153	**0.987**	-0.190	-0.420	0.854	**0.957**	0.521	0.245	0.805	0.154	-0.160	0.131	1							
**Text BC**	-0.87	-0.17	0.617	0.854	-0.810	0.451	-0.850	-0.890	-0.21	0.477	0.929	-0.840	0.276	-0.670	-0.8	-0.660	0.319	1						
**OL BC**	**-0.961**	0.08	0.768	0.948	-0.730	0.632	-0.860	-0.940	0.028	0.629	**0.985**	-0.680	0.510	-0.630	-0.68	-0.480	0.526	**0.966**	1					
**Appe IC**	0.2	-0.01	-0.360	-0.230	-0.380	-0.360	-0.400	-0.260	-0.14	-0.500	0.083	0.047	0.332	-0.620	0.646	0.623	-0.290	-0.14	-0.08	1				
**Smell IC**	0.124	0.27	-0.180	-0.140	-0.220	-0.150	-0.330	-0.250	0.145	-0.300	0.093	0.255	0.536	-0.480	0.742	0.783	-0.05	-0.2	-0.08	**0.959**	1			
**Flavor IC**	-0.94	0.386	**0.957**	**0.954**	-0.260	0.894	-0.470	-0.650	0.399	0.928	0.777	-0.330	0.514	-0.120	-0.700	-0.450	0.810	0.761	0.843	-0.50	-0.39	1		
**Text IC**	-0.83	-0.22	0.617	0.820	-0.680	0.456	-0.700	-0.750	-0.230	0.519	0.828	-0.830	0.119	-0.490	-0.920	-0.810	0.310	**0.968**	0.906	-0.370	-0.440	0.800	1	
**OL IC**	-0.89	-0.15	0.657	0.872	-0.750	0.496	-0.800	-0.860	-0.170	0.532	0.914	-0.820	0.263	-0.600	-0.840	-0.690	0.362	**0.996**	**0.963**	-0.220	-0.280	0.805	**0.984**	1

Coefficients in bold and grey shading indicate a statistically significant correlation ($p<0.05$). Coefficients in bold and blue shading indicate a statistically significant correlation ($p<0.01$). Texture instrumental variables at 50% and 75 % compression (50 and 75 respectively): Hard50 and Hard75 = hardness, Spring50 and Spring75 = springiness, Cohe50 and Cohe75 = cohesiveness, Adh50 and Adh75 = adhesiveness, Chew50 and Chew75 = chewiness, Res50 and Res75 = resilience; Gumm50 and Gumm75 = gumminess. Sensorial consumers: BC = Blind condictions, IC = Informed conditions. Appe= appearance, Text= texture, OL= overall liking.
